# Empagliflozin inhibits coronary microvascular dysfunction and reduces cardiac pericyte loss in db/db mice

**DOI:** 10.3389/fcvm.2022.995216

**Published:** 2022-12-16

**Authors:** Yimin Tu, Qing Li, Yuanchen Zhou, Zixiang Ye, Chao Wu, Enmin Xie, Yike Li, Peizhao Li, Yaxin Wu, Ziyu Guo, Changan Yu, Jingang Zheng, Yanxiang Gao

**Affiliations:** ^1^Department of Cardiology, China-Japan Friendship School of Clinical Medicine, Graduate School of Peking Union Medical College, Chinese Academy of Medical Sciences, Beijing, China; ^2^Department of Cardiology, Peking University China-Japan Friendship School of Clinical Medicine, Beijing, China; ^3^Department of Cardiology, China-Japan Friendship Hospital, Beijing, China

**Keywords:** diabetes, coronary microvascular dysfunction, pericytes, sodium-glucose cotransporter 2 inhibitors, empagliflozin

## Abstract

**Background:**

Coronary microvascular dysfunction (CMD) is a pathophysiological feature of diabetic heart disease. However, whether sodium-glucose cotransporter 2 (SGLT2) inhibitors protect the cardiovascular system by alleviating CMD is not known.

**Objective:**

We observed the protective effects of empagliflozin (EMPA) on diabetic CMD.

**Materials and methods:**

The mice were randomly divided into a db/db group and a db/db + EMPA group, and db/m mice served as controls. At 8 weeks of age, the db/db + EMPA group was given empagliflozin 10 mg/(kg⋅d) by gavage for 8 weeks. Body weight, fasting blood glucose and blood pressure were dynamically observed. Cardiac systolic and diastolic function and coronary flow reserve (CFR) were detected using echocardiography. The coronary microvascular structure and distribution of cardiac pericytes were observed using immunofluorescence staining. Picrosirius red staining was performed to evaluate cardiac fibrosis.

**Results:**

Empagliflozin lowered the increased fasting blood glucose levels of the db/db group. The left ventricular ejection fraction, left ventricular fractional shortening, E/A ratio and E/e′ ratio were not significantly different between the three groups. CFR was decreased in the db/db group, but EMPA significantly improved CFR. In contrast to the sparse and abnormal expansion of coronary microvessels observed in the db/db group, the number of coronary microvessels was increased, and the capillary diameter was decreased in the db/db + EMPA group. The number and microvascular coverage of cardiac pericytes were reduced in the db/db mice but were improved by EMPA. The cardiac fibrosis was increased in db/db group and may alleviate by EMPA.

**Conclusion:**

Empagliflozin inhibited CMD and reduced cardiac pericyte loss in diabetic mice.

## 1 Introduction

Coronary microvascular dysfunction (CMD) refers to abnormalities in the structure and function of the coronary microcirculation that lead to impaired coronary blood flow and a mismatch between myocardial blood supply and oxygen consumption (1, 2). CMD is the pathophysiological basis of various cardiovascular diseases, including diabetic heart disease (3), in which the microvasculature of the diabetic heart shows microaneurysm, sparse capillaries, and basement membrane thickening (4, 5). Recent research revealed that coronary microcirculation disorder caused a fourfold increase in cardiovascular mortality and a fivefold increase in major adverse cardiovascular events (6). There are no effective drugs for the treatment of CMD. Therefore, elucidating the pathological characteristics and mechanisms of CMD is of great significance for preventing and treating cardiovascular diseases.

Pericytes, also known as Rouget cells, are parietal cells distributed on the basement membrane side of endothelial cells in the microvascular system (7) that characteristically express neural glial antigen 2 (NG2), platelet-derived growth factor receptor beta (PDGFRβ) and α-smooth muscle actin (α-SMA) (8). Pericytes play an important role in maintaining vascular stability (9). Under pathological conditions, pericyte injury and apoptosis are associated with breakdown of the vascular wall barrier, activation of the inflammatory response and fibrosis, and promoting the occurrence and development of vascular diseases (10). One of the earliest manifestations of diabetic microvascular complications, such as diabetic retinopathy and diabetic nephropathy, is the loss of pericytes (11). Pericytes are the second most abundant cells in the ventricle and account for 20% of all cells in the heart (8). Pericytes play an important role in regulating myocardial blood flow (12, 13). However, the role of pericytes in diabetic CMD is not clear.

Sodium-glucose cotransporter-2 (SGLT2) inhibitors are a new type of hypoglycemic drug that reduce the reabsorption of glucose by the kidney to exert hypoglycemic effects (14, 15). Several studies revealed that SGLT2 inhibitors reduced blood glucose in diabetic patients and decreased the risk of cardiovascular events (16). Potential mechanisms underlying the cardioprotective effects of SGLT2 inhibitors include improving myocardial energy metabolism (16), alleviating myocardial oxidative stress and fibrosis (17) and reducing myocardial sodium/hydrogen exchange (18). However, whether SGLT2 inhibitors inhibit CMD and its underlying mechanism have not been elucidated.

The present study investigated the features of coronary microcirculation function and structure in type 2 diabetes and pericyte changes in CMD using the db/db mouse model. We treated the diabetic mice with the SGLT2 inhibitor empagliflozin (EMPA) to observe its effects on CMD. The results of this research provide new insights into the prevention and treatment of CMD.

## 2 Materials and methods

### 2.1 Experimental animals

Seven-week-old male db/db mice and littermate db/m mice were purchased from GemPharmatech Co., Ltd. (Nanjing, China). All experimental mice were housed in a 12-h/12-h light/dark cycle room with controlled humidity (50–60%) and temperature (22 ± 2°C) at the Animal Platform of China-Japan Friendship Hospital and provided free access to standard rodent food and water. After adaptive feeding for 1 week, db/db mice were randomly assigned to the db/db group or the db/db + EMPA group. Mice in the db/db + EMPA group were given 10 mg/kg/day EMPA (Boehringer-Ingelheimat, Ingelheim am Rhein, Germany) dissolved in saline intragastrically for 8 weeks, and the mice in the db/m and db/db groups were gavaged with equal amounts of saline. The Institutional Animal Care and Use Committee of China-Japan Friendship Hospital approved all of the animal experiments, which were performed in accordance with all relevant ethical regulations.

### 2.2 Measurement of fasting blood glucose

Mouse fasting blood glucose levels were measured using the hand-held glucometer Accu-Chek Performa (Roche Diabetes Care GmbH) at the following five time points: baseline and 10, 12, 14, and 16 weeks of age. The fasting blood glucose levels in mice were determined from tail snip blood using blood glucose test strips after fasting for 12 h.

### 2.3 Measurement of systolic blood pressure

Systolic blood pressure was measured in conscious mice using a non-invasive tail-cuff device (BP2000 VisiTech International, Sunderland, UK) at 8, 10, 12, 14, and 16 weeks of age. During the procedure, conscious mice were placed in a retainer tube on a warming chamber maintained at 37°C. Mice were acclimatized to the instrument for at least 1 week before baseline measurements were taken. All measurements were performed between 8 and 10 a.m. to avoid variations in blood pressure due to the time of day. Individual mice received 10 initial pressure readings to acclimatize them to the procedure, and 10 additional cycles were measured to obtain the mean systolic pressure. The criteria for data inclusion were the acquisition of at least 10 of 20 measurements and an SD of < 30 mmHg per session.

### 2.4 Urine collection

Mice were placed in metabolic cages for 24 h urine collection within 48 h of the end of treatment.

### 2.5 Echocardiographic evaluation

Echocardiographic measurements were performed in all mice, including coronary microvascular function and cardiac systolic and diastolic function at 8, 10, 12, 14, and 16 weeks of age. We used a high-frequency ultrasound imaging system (Vevo 1100 VisualSonics, Inc., Toronto, ON, Canada) equipped with a 40-MHz central frequency transducer. A single investigator who was blinded to the experimental groups performed all measurements. All parameters were measured at least 3 times, and the means are presented. First, coronary microvascular function assessment was performed. The concentration and duration of isoflurane was referred to the previous study (19). Mice were anesthetized with isoflurane (R510-22-10 RWD Life Science) in a closed chamber with 1% isoflurane in 1 L/min oxygen for 2–5 min until immobile. Each mouse was placed supine on a heated procedure board with isoflurane at 1% supplied by a nose cone connected to the anesthesia machine. Chest hair was removed with chemical cream (Veet, Reckitt Benckiser, London, UK), and ultrasound gel was applied to the chest. The velocity profile in the left coronary artery was monitored for 3 min to ensure that a stable basal signal was achieved, then signals were collected and stored. The isoflurane level was increased to 3% to induce hyperemia, and the velocity profile was monitored for up to 10 min, ensuring the hyperemic CFV becoming maximum and stable. The time signals during this time were stored for analysis of the maximum hyperemic response. Coronary flow reserve was determined as the ratio of hyperemic coronary flow velocity to basal coronary flow velocity.

Cardiac systolic and diastolic function were assessed. The isoflurane concentration was adjusted to approximately 2% to maintain the heart rate of the mice at approximately 400 beats per minute. Diastolic function was obtained using pulsed-wave Doppler from the apical four-chamber view. The isoflurane concentration was reduced to maintain the heart rate at approximately 450–500 beats per minute. Indices of systolic function were obtained from short-axis M-mode scans at the midventricular level, as indicated by the presence of papillary muscles. The following main variables were assessed: left ventricular ejection fraction (LVEF), LV fractional shortening (LVFS), mitral valve (peak E) and maximal peak blood flow levels during mitral atrial systole (peak A) ratio (E/A). The results of E/e′ values were finally measured in 16 weeks of age, which was performed in the Institute of Laboratory Animal Science, CAMS, and PUMC.

### 2.6 Immunostaining

The mice were anesthetized at the conclusion of the experiment. Heparin solution (10 mL/kg, 1000 U/mL) was injected intraperitoneally into the mice. Mice were injected with 10% KCl through the apex until the heart stopped beating, and the right atrial appendage was incised to allow the blood to drain out. The heart and vessels were perfused with a cold heparin-saline solution (10 U/mL) under a constant pressure of 70 mmHg and a 5 mL/min perfusion velocity. When clear fluid flowed out from the right atrial appendage, the heart was perfused with cold 4% paraformaldehyde (PFA) solution for fixation. The hearts were excised from the mice and fixed *via* immersion in 4% PFA solution for 24 h. The hearts were cut transversely into 8 μm (thin) and 150 μm (thick) sections for immunofluorescence staining.

The thick heart slices were sufficiently washed with PBS (2 h * 3) and immersed in 200 μL staining buffer (0.1% w/v sodium azide, 10% Triton X-100, and 0.5% w/v bovine serum albumin in ddH_2_O) with anti-neuroglial cell 2 chondroitin sulfate proteoglycan (NG2) antibody (AB5320, Merck Millipore, Darmstadt, Germany, 1:200) for 3 days at 37°C with gentle shaking. After washing with PBS (2 h * 3), the heart tissues were incubated in 200 μL staining buffer with secondary antibody conjugated to Alexa Fluor 647 (AB32733, Invitrogen, Carlsbad, CA, United States, 1:200) and isolectin B4 conjugated to FITC (L2895, Merck Millipore, Darmstadt, Germany, 1:20) for 3 days at 37°C with gentle shaking. After final washing (PBS, 2 h * 3), the heart slices were attached to glass slides and mounted using antifade reagents with 4′,6-diamidino-2-phenylindole (DAPI) (S36938, Invitrogen, Carlsbad, CA, United States) or a 50% glycerol solution (49767, Sigma, St. Louis, MO, United States).

### 2.7 Laser scanning confocal microscope imaging

Stained sections were imaged using confocal microscopy (Zeiss LSM 800, Leica TCS SP8 DIVE), and 40× objectives (NA = 0.95) were used to visualize cardiac microvessels and pericytes. All images were generated with a 1024 × 1024 pixel frame resolution, and the confocal pinhole was set to 1 AU. For z-stack imaging, the z-step size was set to 1.0 μm to avoid undersampling. At least 6 different fields per sample section were randomly chosen for analysis. The laser power, gain and offset settings were maintained when the same molecule was evaluated in different samples. Images were quantified using ImageJ software. The number of capillaries was counted manually using ImageJ. Ten-micron maximum projection z-stacks were reconstructed for the analysis of capillary diameter, pericyte number and pericyte coverage. The capillary diameter was measured using the Fiji ImageJ “Plot Profile” plug-in analysis tool. The number of NG2-positive perivascular cell bodies that colocalized with DAPI (4′,6-diamidino-2-phenylindole)-positive nuclei was counted using the Fiji ImageJ “ImageJ Cell Counter” plug-in analysis tool to quantify pericytes. The NG2-positive and IB4-positive areas were analyzed using the area analysis module, and pericyte coverage was quantified as the ratio of the NG2 immunostained area to the IB4 immunostained area.

### 2.8 Picrosirius red staining

Picrosirius red staining (PSR) was performed to evaluate cardiac fibrosis. The rehydrated cardiac tissues were stained with Picrosirius red solution (G1018, Servicebio, Wuhan, China) for 8 min. The samples were quickly washed three times with absolute alcohol for 1 min, followed by clearing in xylene. Finally, the specimens were mounted with neutral gum. The picrosirius red–stained sections were scanned with a Pannoramic MIDI slide scanner (3DHISTECH, Budapest, Hungary). The sections were imaged by means of birefringence using polarized light on a microscope (Nikon Eclipse ci, Tokyo, Japan) at ×200 and ×400 magnification. The results of polarized images were red to yellow color for thick fibers and green color for thin fibers. In addition, some part show no polarized effect and appear as black. The percentage of fibrotic area was calculated with the Fiji image J software (20).

### 2.9 Statistical analysis

The results are presented as the means ± SEM. The Shapiro–Wilk test was used to examine the normal distribution of all data. Comparisons between two groups were performed using Student’s t-test for unpaired groups. Comparisons between three groups were performed using one-way or two-way analysis of variance (ANOVA). For multiple comparisons, *p*-values were corrected using the Holm–Bonferroni method. *P* < 0.05 was considered significant. Data analyses were performed using the GraphPad Prism program 8.2.1 version (La Jolla, CA, USA).

## 3 Results

### 3.1 Effects of EMPA on body weight, fasting blood glucose, systolic blood pressure and 24-h urine volume in mice

**FIGURE 1:**
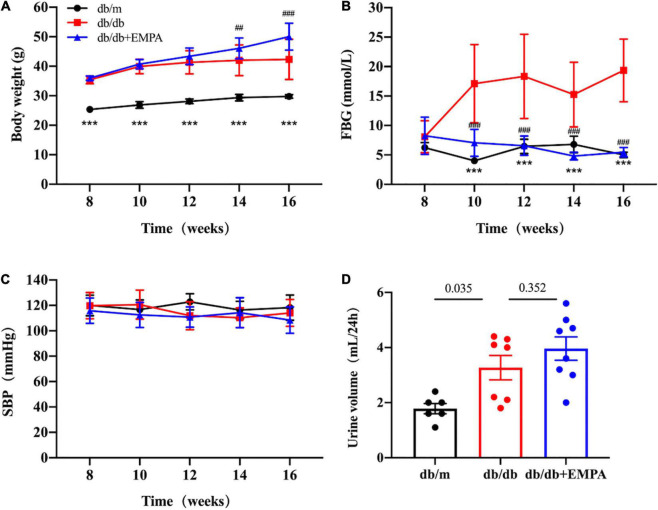
Effects of empagliflozin treatment on basic parameters in different groups. Body weight, fasting blood glucose, urine volume, and systolic blood pressure of mice after different treatments from 8 to 16 weeks of age. Data are presented as the means ± SEM, **(A–C)** the db/m group vs. the db/db group vs. the db/db + EMPA group = 6 vs. 10 vs. 11. **(D)** The db/m group vs. the db/db group vs. the db/db + EMPA group = 6 vs. 7 vs. 8. ***p < 0.001, and the db/m group vs. the db/db group; ^##^*p* < 0.01 and ^###^*p* < 0.001, the db/db + EMPA group vs. the db/db group. FBG, fasting blood glucose; SBP, systolic blood pressure.

From 8 to 16 weeks of age, db/db mice were consistently heavier than db/m mice (Figure 1A). Body weights were initially similar in db/db mice and db/db + EMPA mice. However, mice in the db/db + EMPA group were significantly heavier than db/db mice at 6 and 8 weeks of administration (Figure 1A). At 8 weeks of age, there was no significant difference in fasting blood glucose between the 3 groups (db/m vs. db/db vs. db/db + EMPA: 6.2 ± 0.3 vs. 8.1 ± 0.9 vs. 8.3 ± 1.0 mmol/L, *p* = 0.307) (Figure 1B). However, the fasting blood glucose of db/db mice was significantly higher than db/m mice at 10–16 weeks of age (Figure 1B). From 10 to 16 weeks of age, the fasting blood glucose of the db/db + EMPA group was significantly lower than the db/db mice and similar to the db/m mice (Figure 1B). No significant difference in systolic blood pressure was observed between the 3 groups during the experiment (Figure 1C). At 16 weeks of age, the 24-h urine volume of db/db mice was significantly higher than the db/m mice (3.3 ± 0.4 vs. 1.8 ± 0.2 mL, *p* = 0.035) (Figure 1D), and there was no significant difference between the db/db group and the db/db + EMPA group (4.0 ± 0.4 vs. 3.3 ± 0.4 mL, *p* = 0.352) (Figure 1D).

### 3.2 Effects of EMPA on cardiac function of mice

**FIGURE 2:**
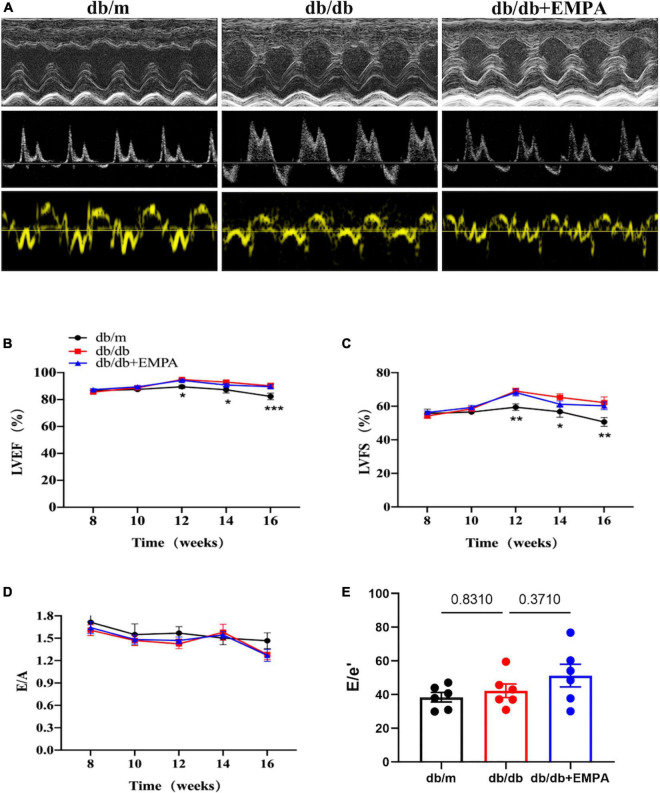
Effects of empagliflozin treatment on cardiac function in db/db mice. **(A)** Representative M-mode (top), pulse-wave Doppler (middle) and Tissue Doppler (bottom) tracings from different experimental groups. **(B)** Left ventricular ejection fraction (LVEF) of different experimental groups over time. **(C)** Left ventricular fractional shortening (LVFS) of different experimental groups over time. **(B,C)** The db/m group vs. the db/db group vs. the db/db + EMPA group = 6 vs. 10 vs. 11. **(D)** E/A ratio of different experimental groups over time, *n* = 6 per group. **(E)** E/e′ ratio of different experimental groups of 16 weeks of age, *n* = 6 per group. Data are represented as the means ± SEM, *p < 0.05, **p < 0.01 and ***p < 0.001 the db/m group vs. the db/db group.

Left ventricular systolic parameters, including the LVEF and LVFS (Figure 2A), increased in the db/db group compared to the db/m group from 12 to 16 weeks of age, but no significant difference was found between the db/db and db/db + EMPA groups (Figures 2B,C). There was no significant difference in E/A and E/e′ between the three groups during treatment (Figures 2D,E).

### 3.3 Empagliflozin inhibited coronary microvascular dysfunction in diabetic mice

**FIGURE 3:**
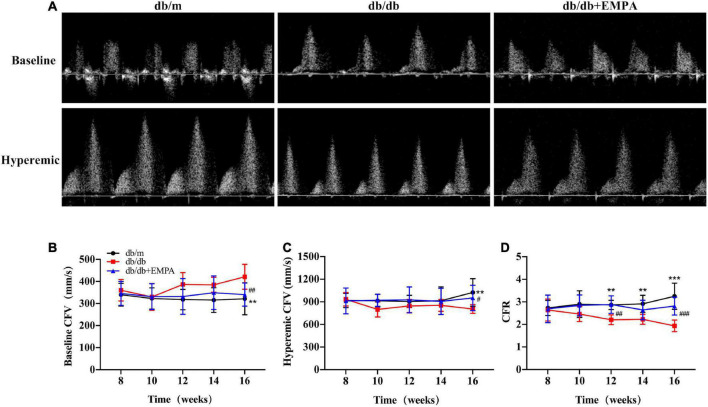
Empagliflozin inhibited coronary microvascular dysfunction in db/db mice. **(A)** Representative images of coronary flow velocity (CFV) recording in the mouse coronary artery. **(B)** Quantitative analyses of baseline CFV of different groups over time. **(C)** Quantitative analyses of hyperemic CFV of different groups over time. **(D)** Quantitative analyses of coronary flow reserve (CFR) of different groups over time. CFR was calculated as the ratio of hyperemic and basal coronary flow velocities. Data are presented as the means ± SEM, the db/m group vs. the db/db group vs. the db/db + EMPA group = 6 vs. 9 vs. 11. **p < 0.01 and ***p < 0.001 the db/m group vs. the db/db group; ^#^*p* < 0.05, ^##^*p* < 0.01 and ^###^*p* < 0.001 the db/db group vs. the db/db + EMPA group.

There was no significant difference in baseline coronary flow velocity (CFV) or hyperemic CFV between the three groups of mice from 8 to 14 weeks of age. At 16 weeks of age, the baseline CFV of db/db mice was significantly higher than the db/m mice (412 ± 2 vs. 321 ± 30 mm/s, *p* = 0.006), and the baseline CFV of the db/db + EMPA group was significantly lower than the db/db group (340 ± 16 vs. 412 ± 2 mm/s, *p* = 0.008). In contrast, the hyperemic CFV of mice in the db/db group was significantly lower than the control group (805 ± 18 vs. 1024 ± 75 mm/s, *p* = 0.012), and the hyperemic CFV in the db/db + EMPA group was higher than the db/db group (952 ± 51 vs. 805 ± 18 mm/s, *p* = 0.048). However, the CFR showed a significant difference between the 3 groups from 12 weeks of age. At 16 weeks of age, the CFR of the db/db group was significantly lower than the control group (1.9 ± 0.1 vs. 3.3 ± 0.2, *p* < 0.001), and the CFR of the db/db + EMPA group was higher than the db/db group (2.8 ± 0.1 vs. 1.9 ± 0.1, *p* < 0.001) (Figure 3).

### 3.4 Empagliflozin ameliorated abnormal microvascular structure in the hearts of diabetic mice

**FIGURE 4:**
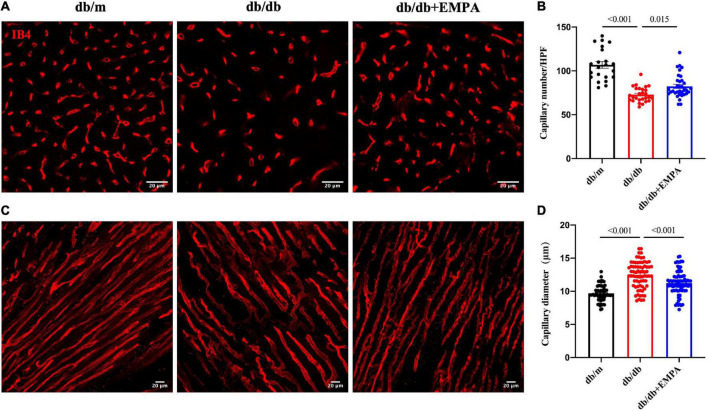
Effects of empagliflozin treatment on the cardiac microvasculature of db/db mice. **(A)** Representative transverse images of myocardial vasculature showing capillaries (IB4+, red). **(B)** Statistical analysis of the density of capillaries in the transverse sections of myocardial tissue between the db/m group, db/db group, and db/db + EMPA group. **(C)** Representative longitudinal images of myocardial vasculature showing capillaries (IB4+, red). **(D)** Statistical analysis of the diameter of capillaries in the longitudinal sections of myocardial tissue between the db/m group, db/db group, and db/db + EMPA group. Data are presented as the means ± SEM. *P*-values are corrected for multiple comparisons. IB4, isolectin B4; HPF, high power field.

Microvessels were labeled with IB4, and higher magnification images of thick heart slices were acquired to assess the coronary microvascular structure of the mice (Figures 4A,C) and calculated per high power field (HPF). The number of cardiac capillaries was significantly reduced in db/db mice compared to controls (73 ± 2/HPF vs. 107 ± 4/HPF, *p* < 0.001), and the cardiac capillary density of db/db + EMPA mice was higher than the db/db mice (82 ± 2/HPF vs. 73 ± 2/HPF, *p* = 0.015) (Figure 4B). The diameter of cardiac capillaries was significantly increased in db/db mice compared to controls (12.5 ± 0.2 vs. 9.7 ± 0.1 μm, *p* < 0.001), and the capillary diameter was decreased in db/db + EMPA mice compared to db/db mice (11.3 ± 0.2 vs. 12.5 ± 0.2 μm, *p* < 0.001) (Figure 4D). In summary, EMPA ameliorated the abnormal dilation and sparseness of cardiac capillaries in db/db mice.

### 3.5 Empagliflozin inhibited pericyte loss in the hearts of diabetic mice

**FIGURE 5:**
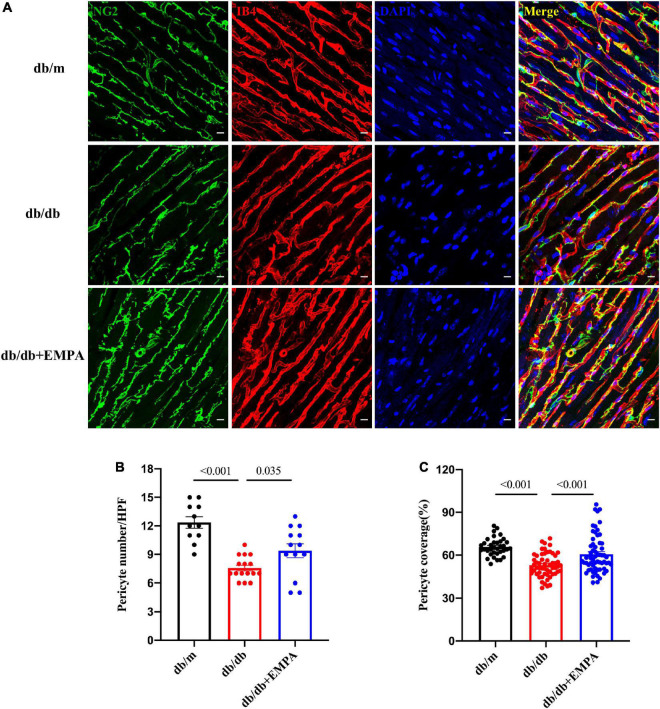
Effects of empagliflozin treatment on pericytes in the hearts of db/db mice. **(A)** Representative myocardial sections showing pericytes (NG2+, green), capillaries (IB4+, red) and nuclei (DAPI+, blue). **(B)** Statistical analysis of pericyte number/HPF between the three groups. **(C)** Statistical analysis of pericyte coverage between the three groups, pericyte coverage = (NG2 + area/IB4 + area)/HPF*100%. Data are presented as the means ± SEM. *P*-values are corrected for multiple comparisons. NG2, neuroglial cell 2 chondroitin sulfate proteoglycan; IB4, isolectin B4; DAPI, 4′,6-diamidino-2-phenylindole; HPF, high power field.

Pericytes were labeled with an anti-NG2 antibody, and higher magnification images of thick heart slices were acquired to assess the role of pericytes in the coronary microcirculation. The number of pericytes was significantly reduced in db/db mice compared to the controls (7.6 ± 0.3/HPF vs. 12.6 ± 0.6/HPF, *p* < 0.001), and that of db/db + EMPA mice was higher than db/db mice (9.4 ± 0.7/HPF vs. 7.6 ± 0.3/HPF, *p* = 0.035). Pericyte coverage, represented as the ratio of the NG2-positive area to the IB4-positive area (Figure 5A), was significantly decreased in the hearts of the db/db group (53 ± 1% vs. 66 ± 1%, *p* < 0.001). Cardiac pericyte coverage was higher in db/db + EMPA mice than db/db mice (61 ± 2% vs. 53 ± 1%, *p* < 0.001) (Figures 5B,C). Overall, EMPA inhibited cardiac pericyte loss and increased the pericyte coverage of the coronary microvasculature in diabetic mice.

### 3.6 Empagliflozin improved cardiac fibrosis in the hearts of diabetic mice

Picrosirius red staining and polarized light microscopy were used to determine the cardiac fibrosis between 3 groups. Representative images of PSR staining illustrating different collagen levels were shown in Supplementary Figure 1A. Subsequent image processing enabled measuring the percentage of positive area per image for PSR. This suggested the collagen in myocardium increased in db/db mice compared with db/m mice (0.35 ± 0.14 vs. 0.98 ± 0.39%, *p* = 0.009), and was improved in db/db + EMPA mice (0.98 ± 0.39% vs. 0.59 ± 0.19%, *p* = 0.0191) (Supplementary Figure 1B). In db/db group, the myocardial fibrosis was severer than the other groups, which might lead to the cardiac stiffness and be improved by EMPA.

## 4 Discussion

The present study observed the functional and structural characteristics of the coronary microcirculation in db/db mice and the effects of EMPA on the coronary microcirculation in db/db mice using echocardiography, immunofluorescence staining and confocal laser. The results showed that coronary blood flow reserve decreased in db/db mice, the number of cardiac capillaries was reduced, and cardiac capillaries were abnormally dilated. The number and coverage of cardiac pericytes also decreased. The results of PSR revealed EMPA might decrease the cardiac collagen caused by diabetes. Empagliflozin ameliorated the functional and structural abnormalities of the coronary microcirculation and inhibited the decrease in cardiac pericyte number and coverage in db/db mice. These results indicated that cardiac pericyte loss was related to CMD, and EMPA ameliorated cardiac pericyte loss and alleviated CMD in type 2 diabetes.

Our study showed that db/db mice had mildly increased cardiac systolic function from 12 to 16 weeks of age without any significant change in diastolic function. However, the coronary microvascular function index CFR decreased significantly at 12 weeks of age in db/db mice, and the coronary microvessels were sparse and dilated at 16 weeks of age. These results suggest that coronary microvascular function and structural disturbances in db/db mice appear before cardiac systolic and diastolic dysfunction. Therefore, CMD is one of the early pathological manifestations of diabetic heart disease. Guimbal et al. (21) observed the structure and function of cardiac microvessels in 12-week-old female db/db mice and found that cardiac microvessel density was reduced. In a prospective cohort study, impaired CFR was independently associated with diastolic dysfunction and adverse events, especially HFpEF hospitalization in symptomatic patients without overt coronary artery disease (22). Microvascular rarefaction might precede disease development, as HFpEF-associated comorbidities, type 2 diabetes mellitus, aging, hypertension, and obesity, show microvascular rarefaction (23). For example, microvascular rarefaction is suggested to impede insulin delivery to muscles and adipose tissue, contributing to poor insulin uptake (24). Furthermore, increased subepicardial and pericoronary adipose tissue, as observed in obese, T2DM, and elderly patients, correlated with an impaired CFR, microvasculature, and coronary function, leading to deteriorated diastolic function (25–27). Our observations corroborate and extend these findings by suggesting that CMD plays an important role in the development of diabetic heart disease.

Single-cell sequencing results revealed that there were many pericytes in the human heart (8). Increasing evidence indicates that pericytes perform diverse functions, such as promoting angiogenesis, maintaining vascular stability, regulating blood flow, forming the blood-brain barrier/blood-retina barrier, and regulating neuroinflammation (9). Previous studies showed that pericyte abnormalities played an important role in microvascular diseases (28). For example, after coronary ischemia-reperfusion in rats, the reduction in cardiac capillary perfusion in the ischemic region was associated with pericyte contraction (29). Pericyte loss is a well-established hallmark of diabetic retinopathy (30) and diabetic heart disease in patients (31). Our study found that the number and coverage of pericytes in the hearts of db/db mice were significantly reduced and coexisted with functional and structural abnormalities of coronary microcirculation in diabetic mice. Therefore, pericyte loss may be an important factor in the development of diabetic CMD.

Although evidence from large clinical trials is lacking, EMPA improved coronary microvascular function in diabetic mice in preclinical studies (32). Another study showed that empagliflozin prevented coronary microangiopathy in mice with type 2 diabetes by acting on sGC-cGMP-PKG (33). The effect of empagliflozin on cardiac microvascular endothelial cell-mediated preservation of cardiomyocyte function suggests that empagliflozin could be used to treat the cardiac mechanical implications of microvascular dysfunction in HFpEF (34). Kidney and retinal pericytes express SGLT2, and SGLT2 inhibitors inhibit high glucose-induced pericyte swelling (35). A recent study showed that a low-dose SGLT2 inhibitor ameliorated ischemic brain injury in mice *via* pericyte protection without glucose-lowering effects (36). Our study found that EMPA inhibited cardiac pericyte loss and ameliorated CMD in type 2 diabetic mice. The protective effects of EMPA on pericytes may play an important role in the treatment of coronary microvascular diseases, such as diabetic CMD.

In diabetic subjects, fibrotic expansion of the cardiac interstitium assessed through MRI is also associated with adverse outcome (37). HFpEF patients exhibit prominent interstitial myocardial fibrosis, associated with coronary microvascular rarefaction (38). Pericyte loss or dysfunction is commonly observed in diverse fibrotic diseases, such as cardiac fibrosis (39), diabetic retinopathy, and neurodegenerative diseases (40). Several cell types, including pericytes, were implicated in fibrotic remodeling of the heart, either by secreting fibrogenic mediators and matricellular proteins, or in some cases, by undergoing conversion to activated myofibroblasts. It is interesting to investigate the association and mechanism between pericytes loss and cardiac fibrosis. The analysis of the picrosirius red staining pictures in our study revealed that cardiac fibrosis might be improved by EMPA. This result was consistent with the previous study (17).

The current study has some limitations that should be addressed in future research. First, the contribution of SGLT2 inhibitors in alleviating pericyte loss and CMD must be further explored. Previous studies showed that a high glucose state induced pericyte apoptosis, migration, or differentiation into other types of cells. It is necessary to investigate the expression of SGLT2 in the heart and examine whether SGLT2 inhibitors directly act on the cells that constitute the coronary microcirculation, such as pericytes and endothelial cells, or indirectly protect the coronary microcirculation by lowering blood glucose. CMD, in the absence of appreciable atherosclerosis, was severe enough to produce perturbations in myocardial oxygen balance (41), which might affect the pericytes loss. *In vivo* data of diabetic retinopathy showed that the early disappearance of pericytes was quickly followed by the loss of ECs and capillary network collapse, leading to reduced blood flow in the retina (42). Therefore, these speculations of cardiac pericyte loss are promising research directions. Second, 16-week-old male db/db mice showed no abnormal cardiac systolic or diastolic function in our study, and EMPA had no significant effect on cardiac function. An extended experiment may clarify the relationship between diabetic CMD and abnormal cardiac function and whether EMPA improved cardiac systolic or diastolic function by protecting the coronary microcirculation. Third, the use of CFR based on flow velocity rather than volumetric flow per gram of myocardial tissue. CFR measurement is now recommended by international guidelines (43) as a diagnostic method for the identification of patients with microvascular angina who could benefit from targeted therapy (44). In INOCA patients with functional CMD (45), in patients with residual CMD after undergoing percutaneous coronary intervention for obstructive CAD (46), and in patients with diabetes mellitus (47), an increase in basal coronary blood flow per gram of myocardium (45) or increases in basal coronary flow velocity (46, 47), as compared to healthy individuals, appears primarily responsible for the reduction in CFR. Forth, the lack of measurement of arterial blood pressure at the time of CFR measurements, preventing the assessment of coronary vascular conductance/resistance. In our laboratory, it’s difficult for us to measure blood pressure and CFR at the same time. Because the non-invasive tail-cuff device and echocardiographic device were separate. However, before CFR was measured per 2 weeks, arterial blood pressure was measured. Blood pressure was not significantly different in the 3 groups from 8 to 16 weeks of age. Therefore, the CFR should not be influenced by blood pressure. We will examine these issues in future studies.

## 5 Conclusion

Abnormal coronary microvascular structure and function occur in the early stage of diabetic heart disease. The decreased number and coverage of cardiac pericytes may be involved in the progression of CMD. Empagliflozin attenuated coronary microvascular function and structural abnormalities and protected cardiac pericytes in diabetic mice. Therefore, EMPA may be an effective drug for the treatment of diabetic CMD.

## Data availability statement

The original contributions presented in this study are included in the article/Supplementary material, further inquiries can be directed to the corresponding author/s.

## Ethics statement

The animal study was reviewed and approved by Institutional Animal Care and Use Committee of China-Japan Friendship Hospital.

## Author contributions

YT: validation, methodology, visualization, writing—original draft, and writing—review and editing. QL: methodology, visualization, and data curation. YZ: data curation, software, and writing—review and editing. ZY: visualization, software, data curation, and writing—review and editing. CW and EX: data analysis and writing—review and editing. YL, PL, and YW: methodology and writing–review and editing. ZG and CY: visualization, software, and writing–review and editing. JZ: conceptualization, methodology, investigation, resources, data curation, visualization, supervision, funding acquisition, and writing—review and editing. YG: conceptualization, investigation, resources, data curation, supervision, project administration, visualization, and writing—review and editing. All authors contributed to the article and approved the submitted version.
