# Supplementary material for: Empagliflozin inhibits coronary microvascular dysfunction and reduces cardiac pericyte loss in db/db mice
Source: Front Cardiovasc Med. 2022 Dec 16;9:995216. doi: 10.3389/fcvm.2022.995216 (PMC9800791; doi:10.3389/fcvm.2022.995216)
Supplement: Supplementary file 1 [file Table_1.DOCX]

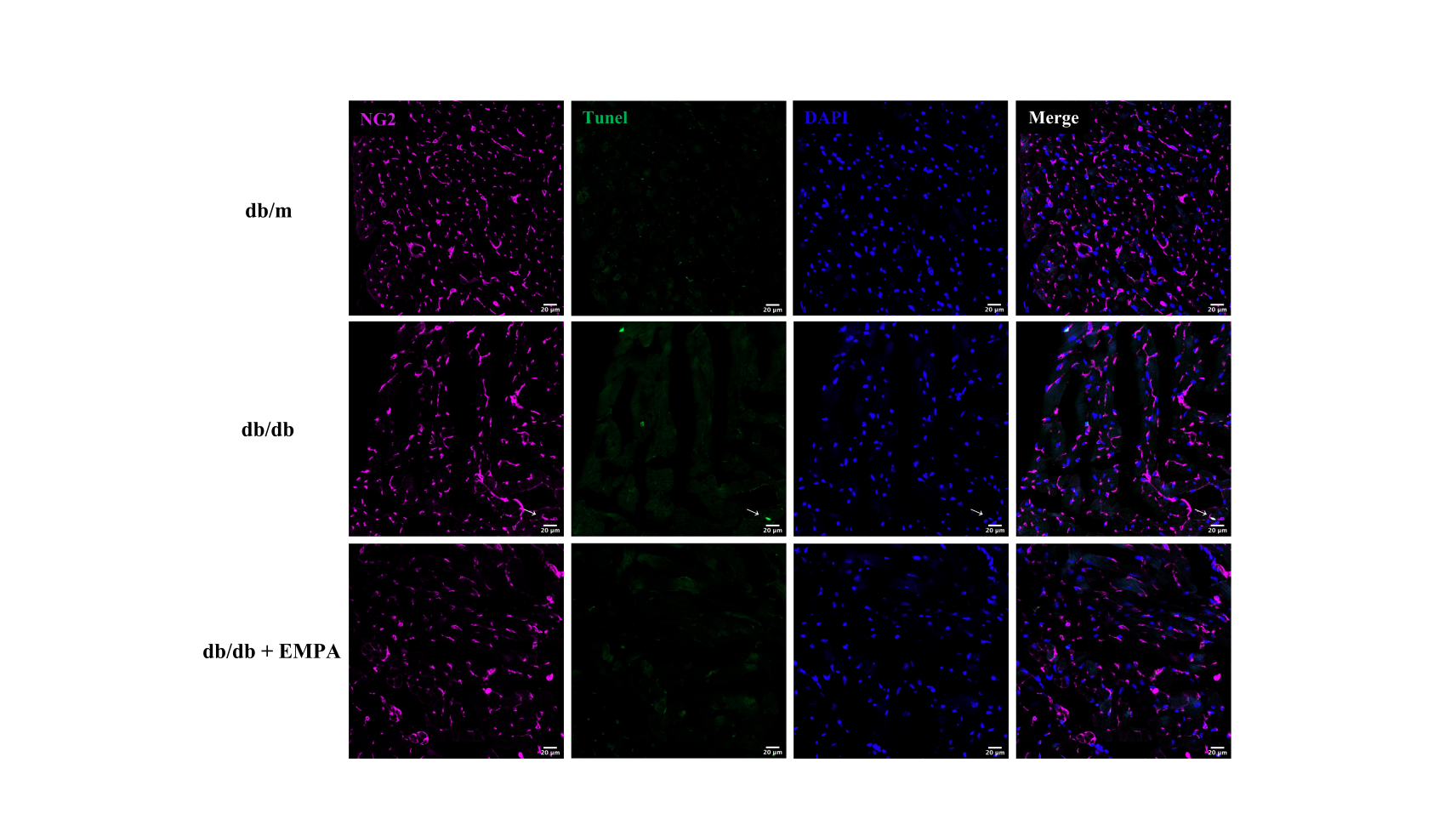


Cardiac pericytes apoptosis of db/m, db/db and db/db+EMPA mice at the age of 16 weeks. Representative myocardial sections showing pericytes (NG2+, purple), apoptotic cell (TUNEL+, green) and nuclei (DAPI+, blue). Apoptotic cells, white arrow. Abbreviation: NG2, neuroglial cell 2 chondroitin sulfate proteoglycan; TUNEL, TdT-mediated dUTP Nick-End Labeling; DAPI, 4’,6-diamidino-2-phenylindole.
