# Supplementary material for: Empagliflozin inhibits coronary microvascular dysfunction and reduces cardiac pericyte loss in db/db mice
Source: Front Cardiovasc Med. 2022 Dec 16;9:995216. doi: 10.3389/fcvm.2022.995216 (PMC9800791; doi:10.3389/fcvm.2022.995216)
Supplement: Supplementary file 2 [file Table_2.DOCX]

Supplementary Material

# Supplementary Figures and Tables

## Supplementary Figures


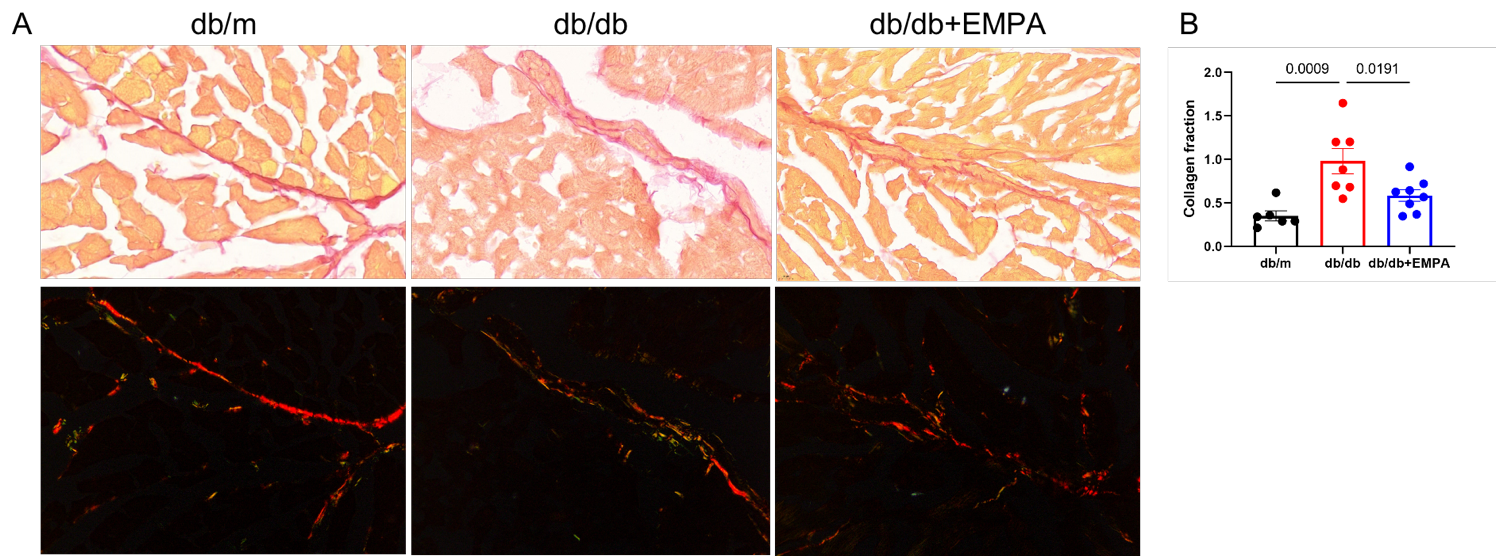


**Supplementary Figure 1.** **Empagliflozin suppresses myocardial fibrosis in db/db mice.** A. Representative Picrosirius red stained heart sections of different experimental groups imaged under light microscopy and polarized microscopy taken at 40× magnification. B. The percentages of positive areas of collagen fraction. Data are expressed as the mean ± SEM.
