# Supplementary figures and images for: Empagliflozin inhibits coronary microvascular dysfunction and reduces cardiac pericyte loss in db/db mice
Source: Front Cardiovasc Med. 2022 Dec 16;9:995216. doi: 10.3389/fcvm.2022.995216 (PMC9800791; doi:10.3389/fcvm.2022.995216)

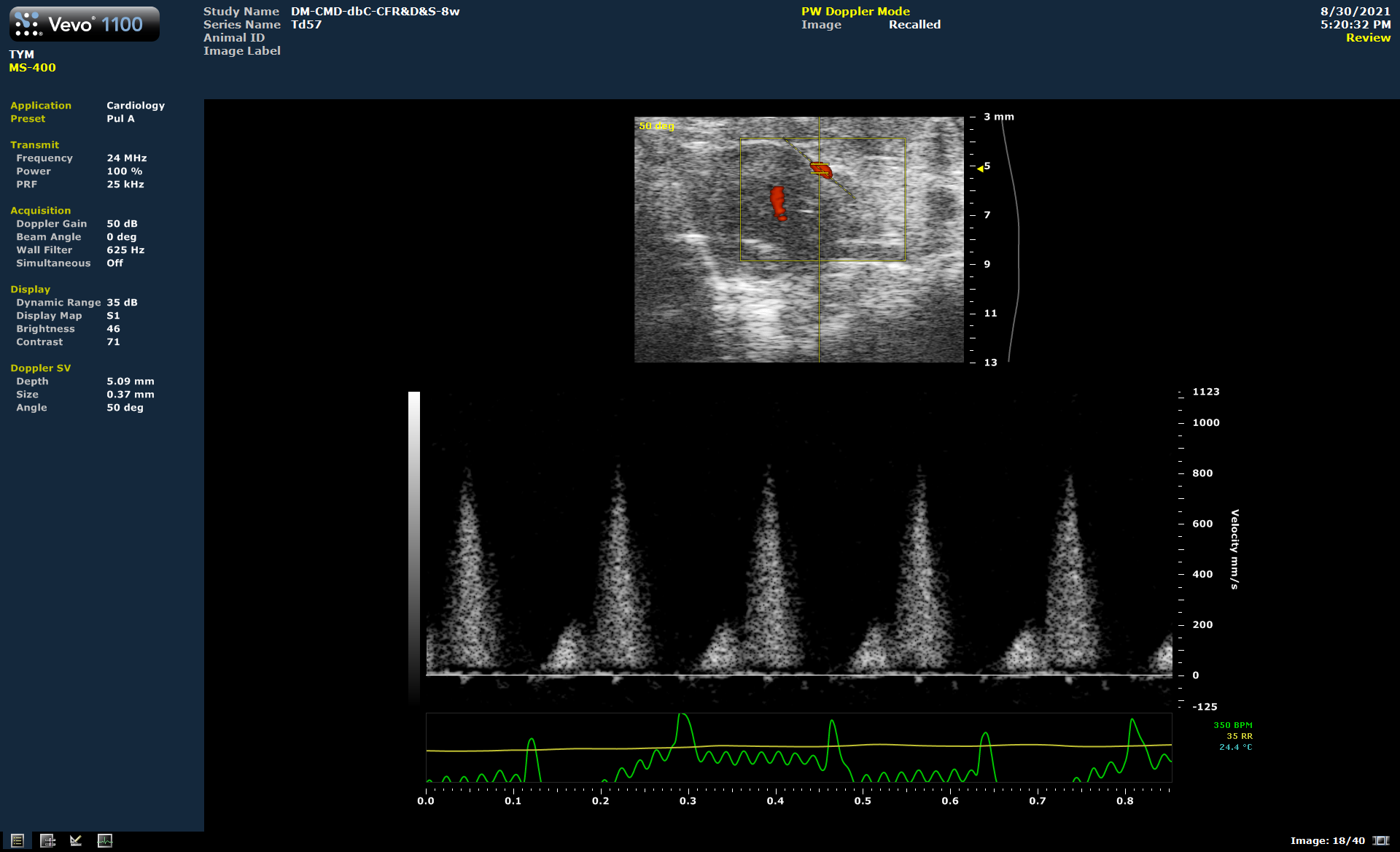

Supplement: Supplementary file 3 [file Data_Sheet_1.ZIP › 0801σÄƒσoïμò░μì«20221124Σ┐«μö╣/Figure3/db:db-9-hyperemic CFV.tif]

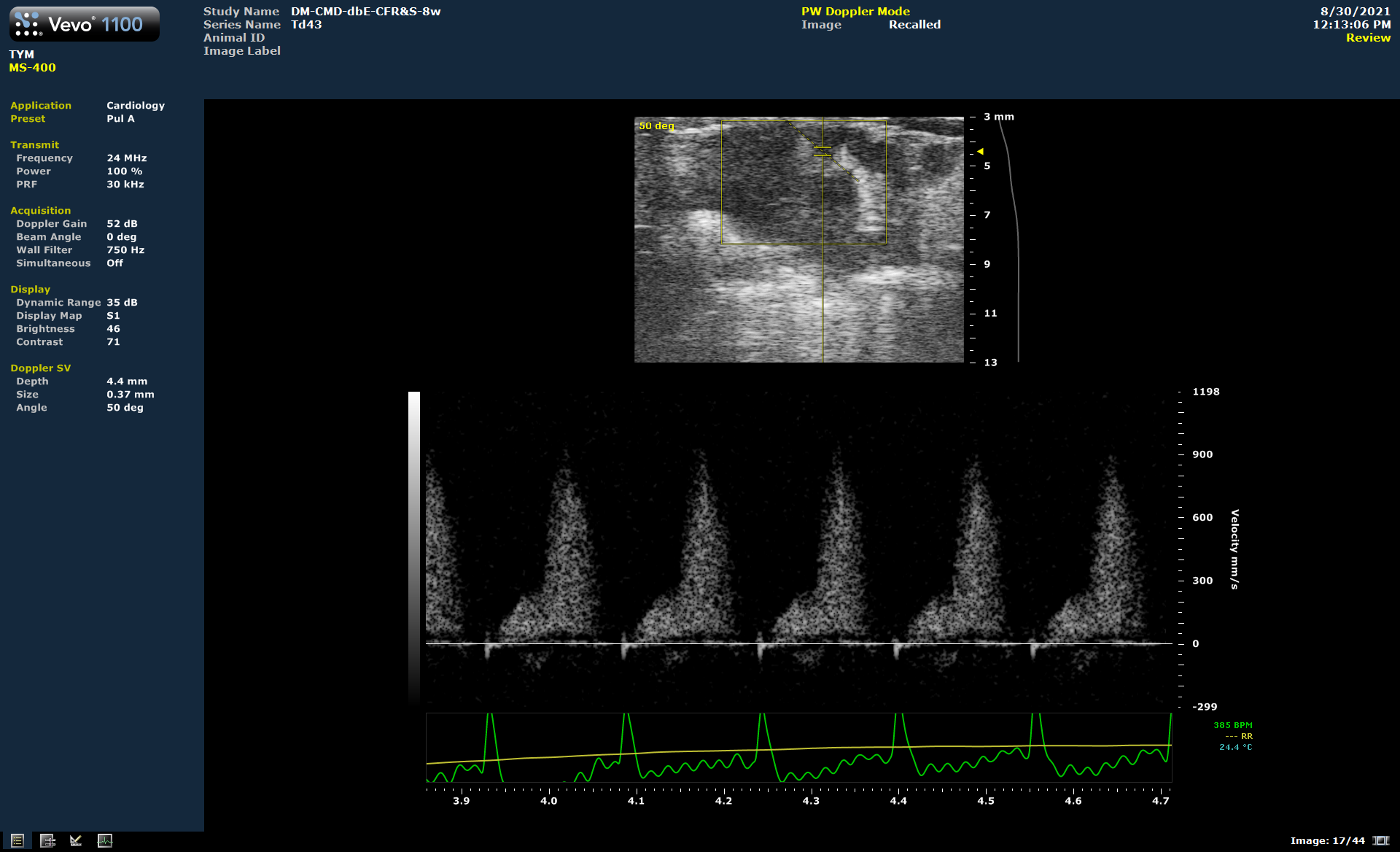

Supplement: Supplementary file 3 [file Data_Sheet_1.ZIP › 0801σÄƒσoïμò░μì«20221124Σ┐«μö╣/Figure3/db:db+EMPA-1-hyperemic CFV.tif]

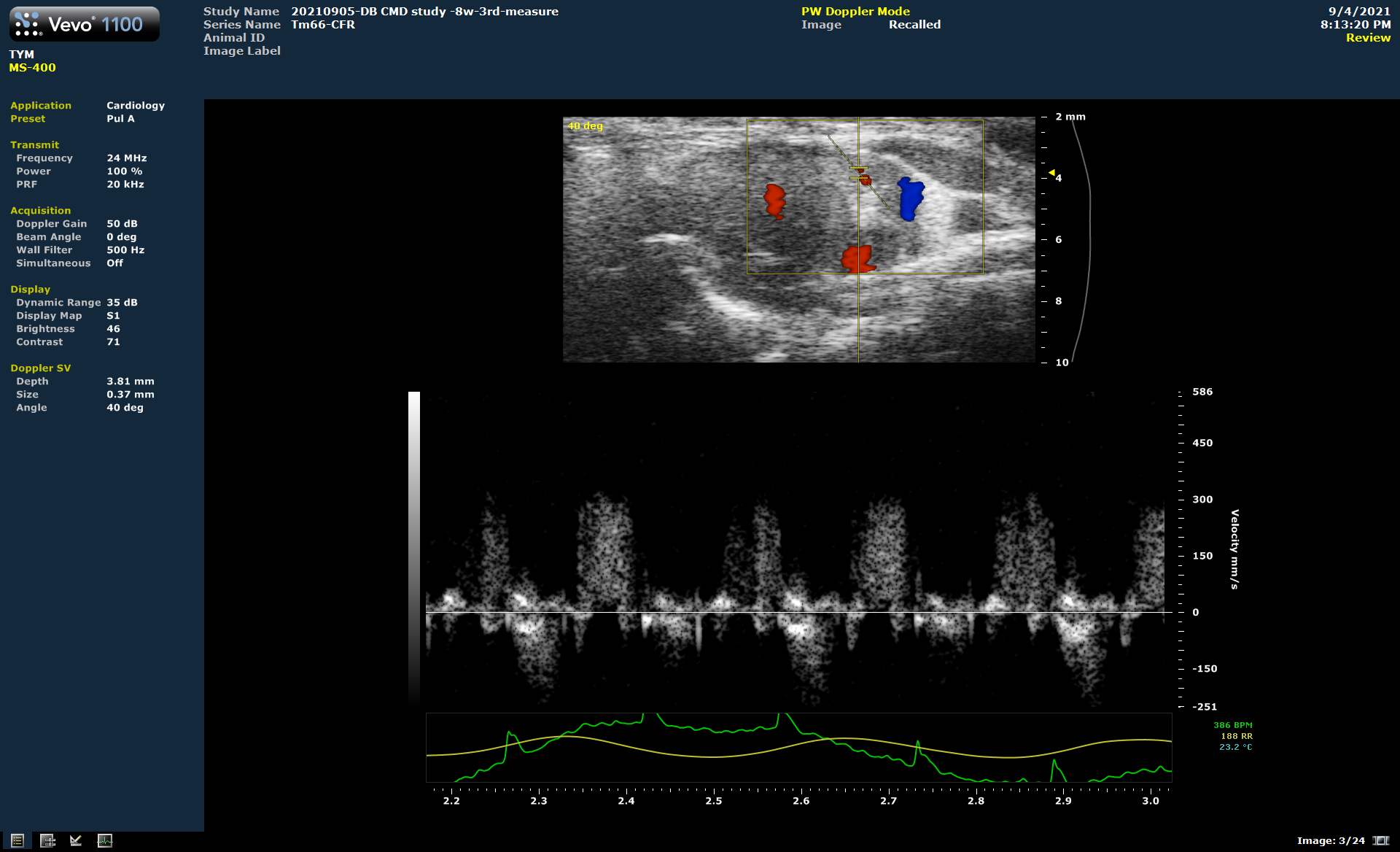

Supplement: Supplementary file 3 [file Data_Sheet_1.ZIP › 0801σÄƒσoïμò░μì«20221124Σ┐«μö╣/Figure3/db:m-5-baseline CFV.tif]

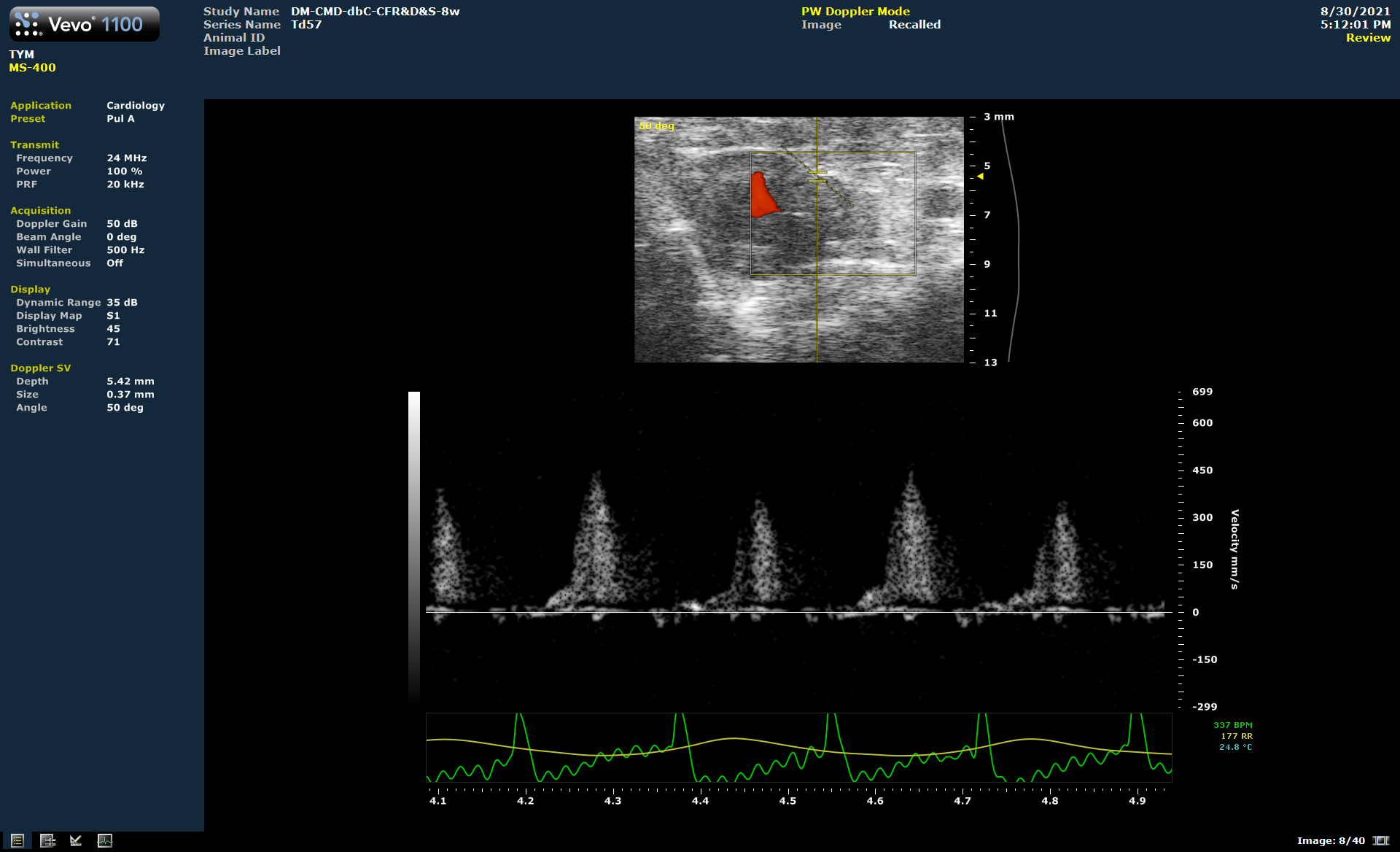

Supplement: Supplementary file 3 [file Data_Sheet_1.ZIP › 0801σÄƒσoïμò░μì«20221124Σ┐«μö╣/Figure3/db:db-9-baseline CFV.tif]

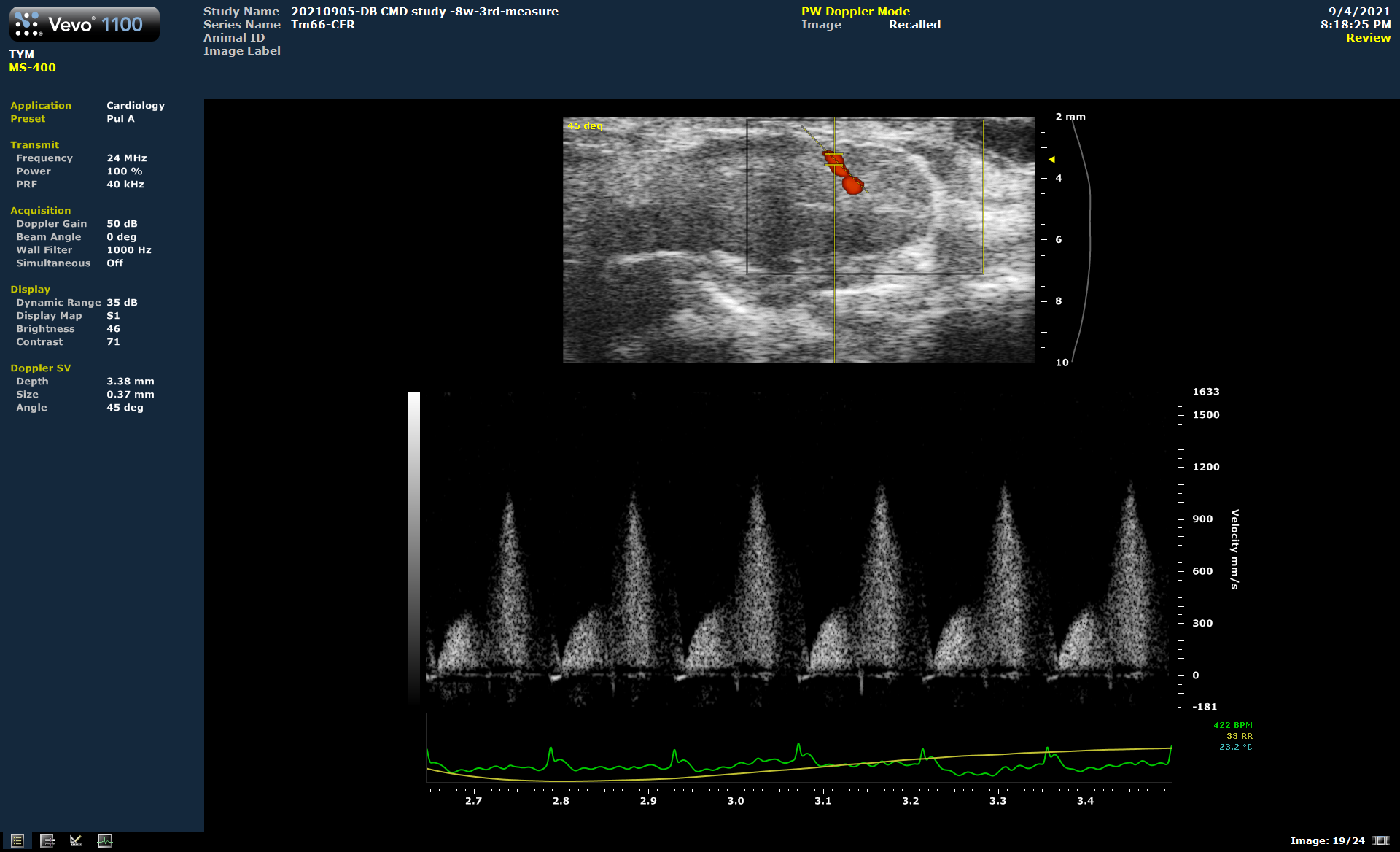

Supplement: Supplementary file 3 [file Data_Sheet_1.ZIP › 0801σÄƒσoïμò░μì«20221124Σ┐«μö╣/Figure3/db:m-5-hyperemic CFV.tif]

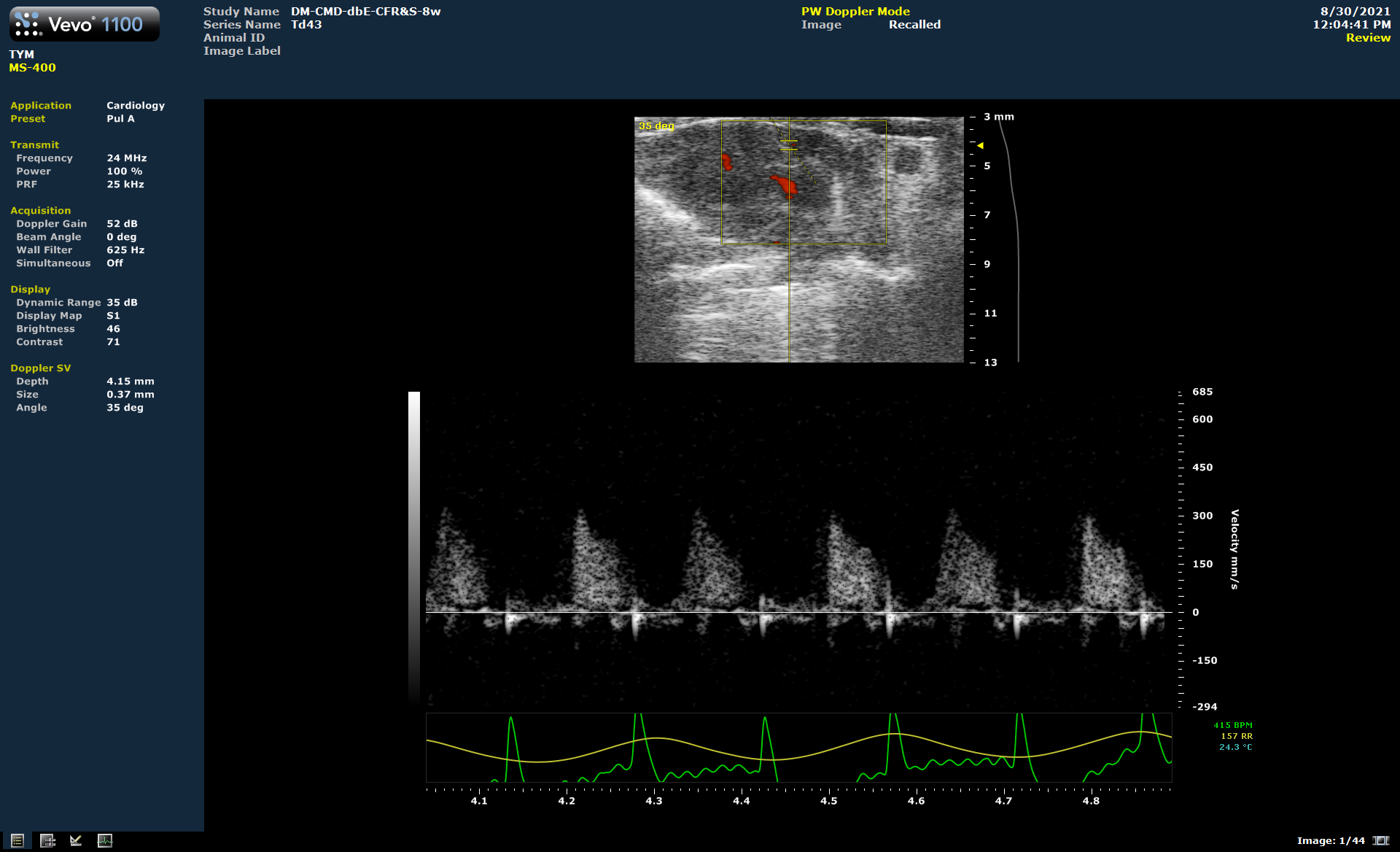

Supplement: Supplementary file 3 [file Data_Sheet_1.ZIP › 0801σÄƒσoïμò░μì«20221124Σ┐«μö╣/Figure3/db:db+EMPA-1-baseline CFV.tif]

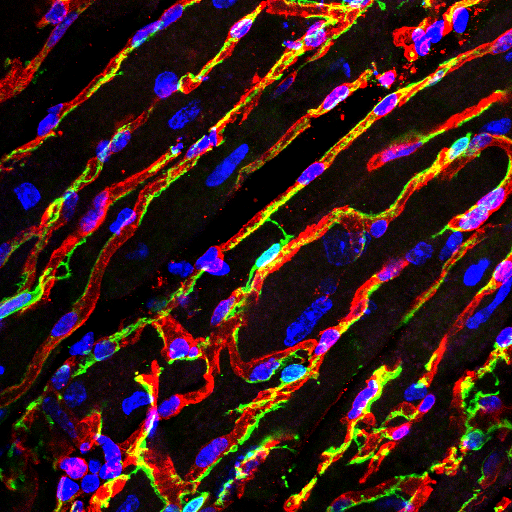

Supplement: Supplementary file 3 [file Data_Sheet_1.ZIP › 0801σÄƒσoïμò░μì«20221124Σ┐«μö╣/Figure5/db:db/db:db-10-Merge-x40.tif]

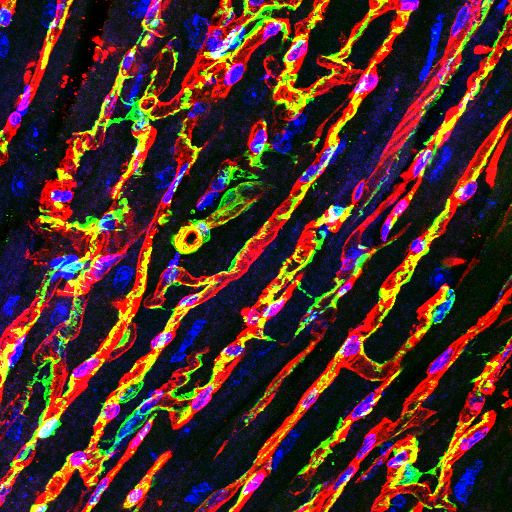

Supplement: Supplementary file 3 [file Data_Sheet_1.ZIP › 0801σÄƒσoïμò░μì«20221124Σ┐«μö╣/Figure5/db:db+EMPA/db:db+EMPA-10-Merge-x40.tif]

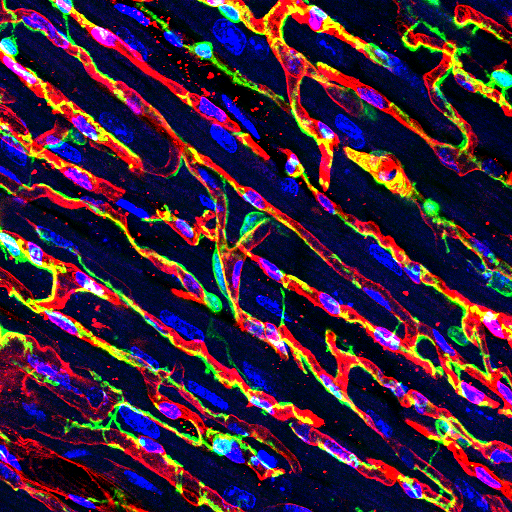

Supplement: Supplementary file 3 [file Data_Sheet_1.ZIP › 0801σÄƒσoïμò░μì«20221124Σ┐«μö╣/Figure5/db:m/db:m-5-Merge-x40.tif]

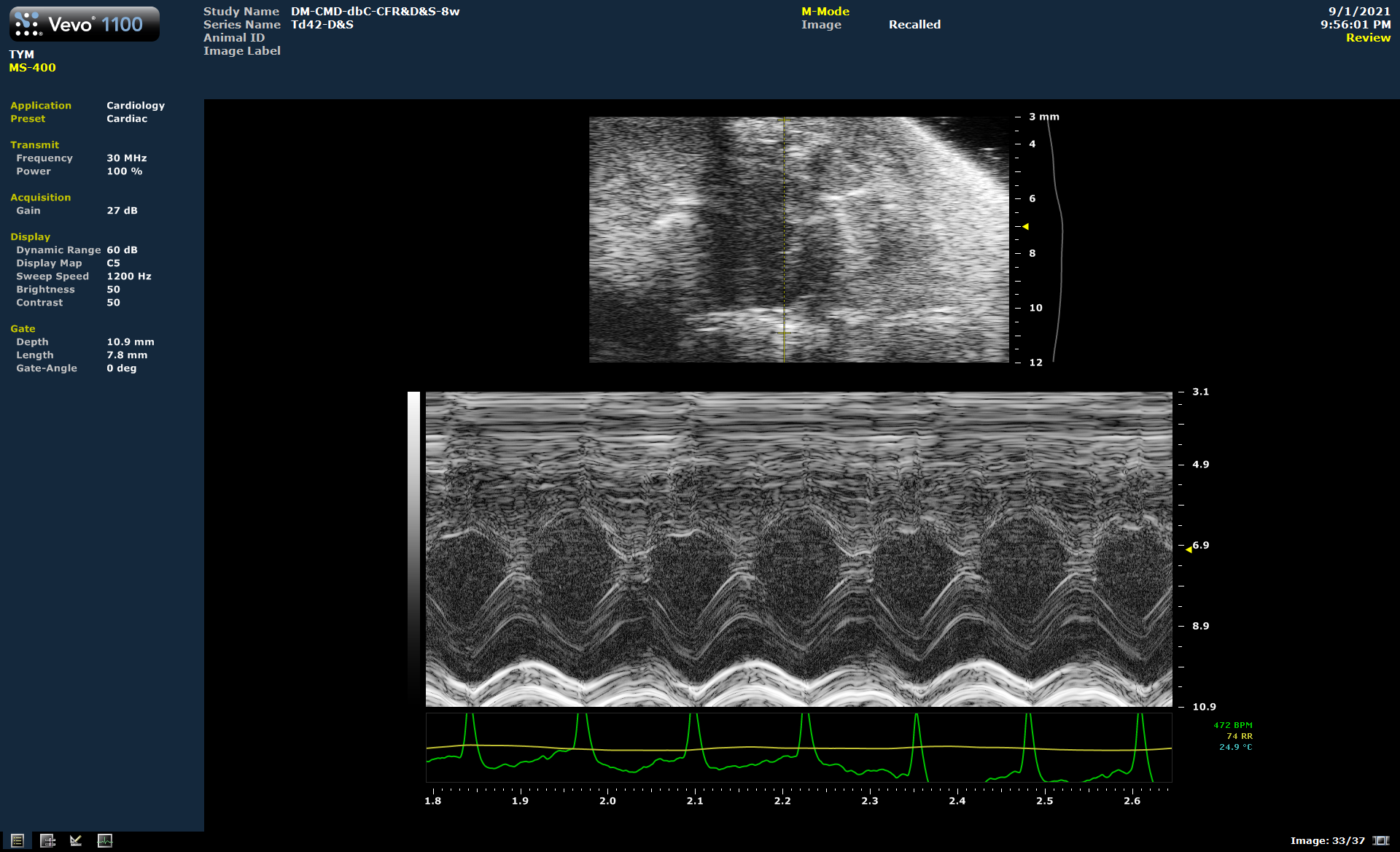

Supplement: Supplementary file 3 [file Data_Sheet_1.ZIP › 0801σÄƒσoïμò░μì«20221124Σ┐«μö╣/Figure2/Cardiac systolic function/db:db-2-systolic.tif]

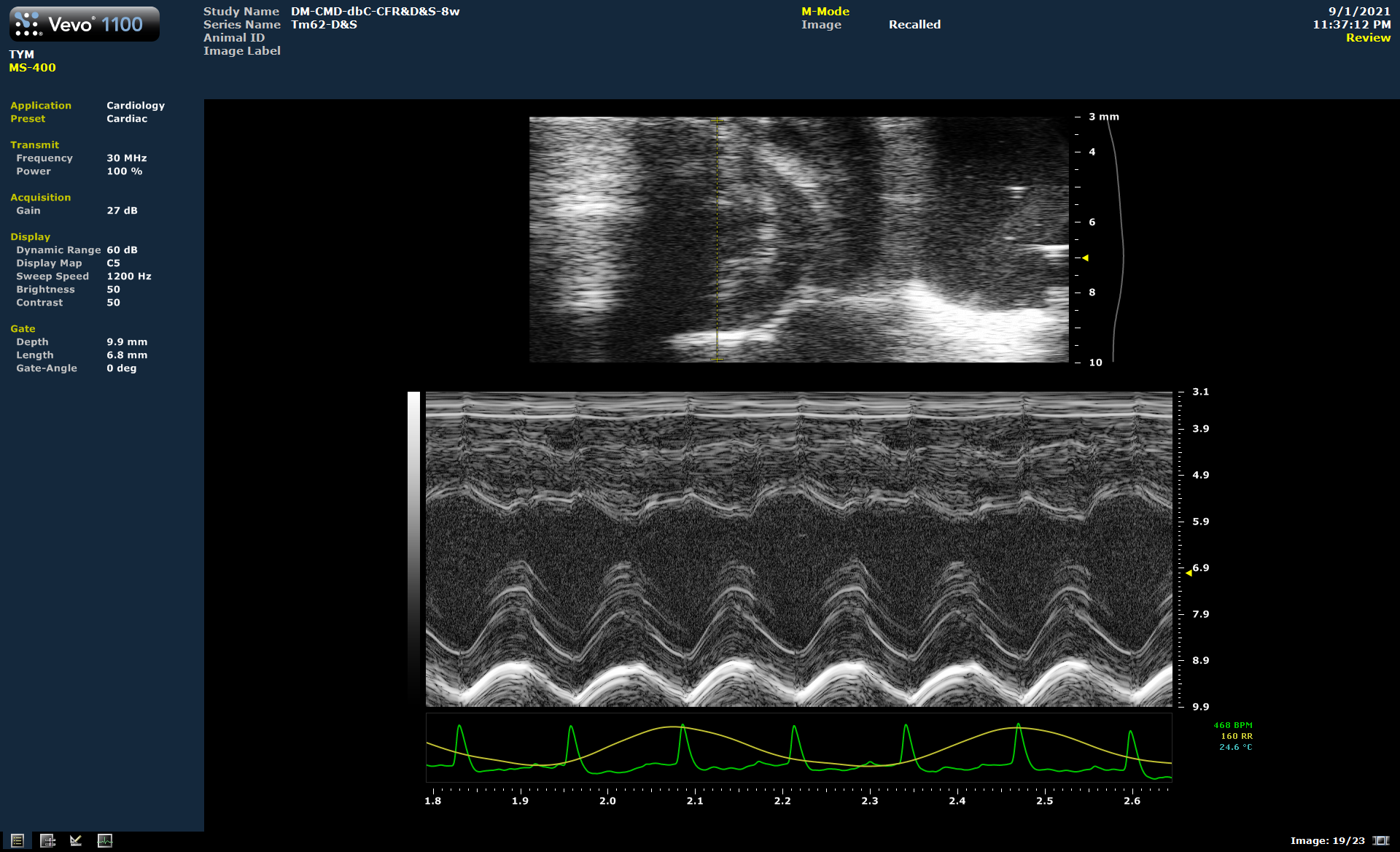

Supplement: Supplementary file 3 [file Data_Sheet_1.ZIP › 0801σÄƒσoïμò░μì«20221124Σ┐«μö╣/Figure2/Cardiac systolic function/db:m-1-systolic.tif]

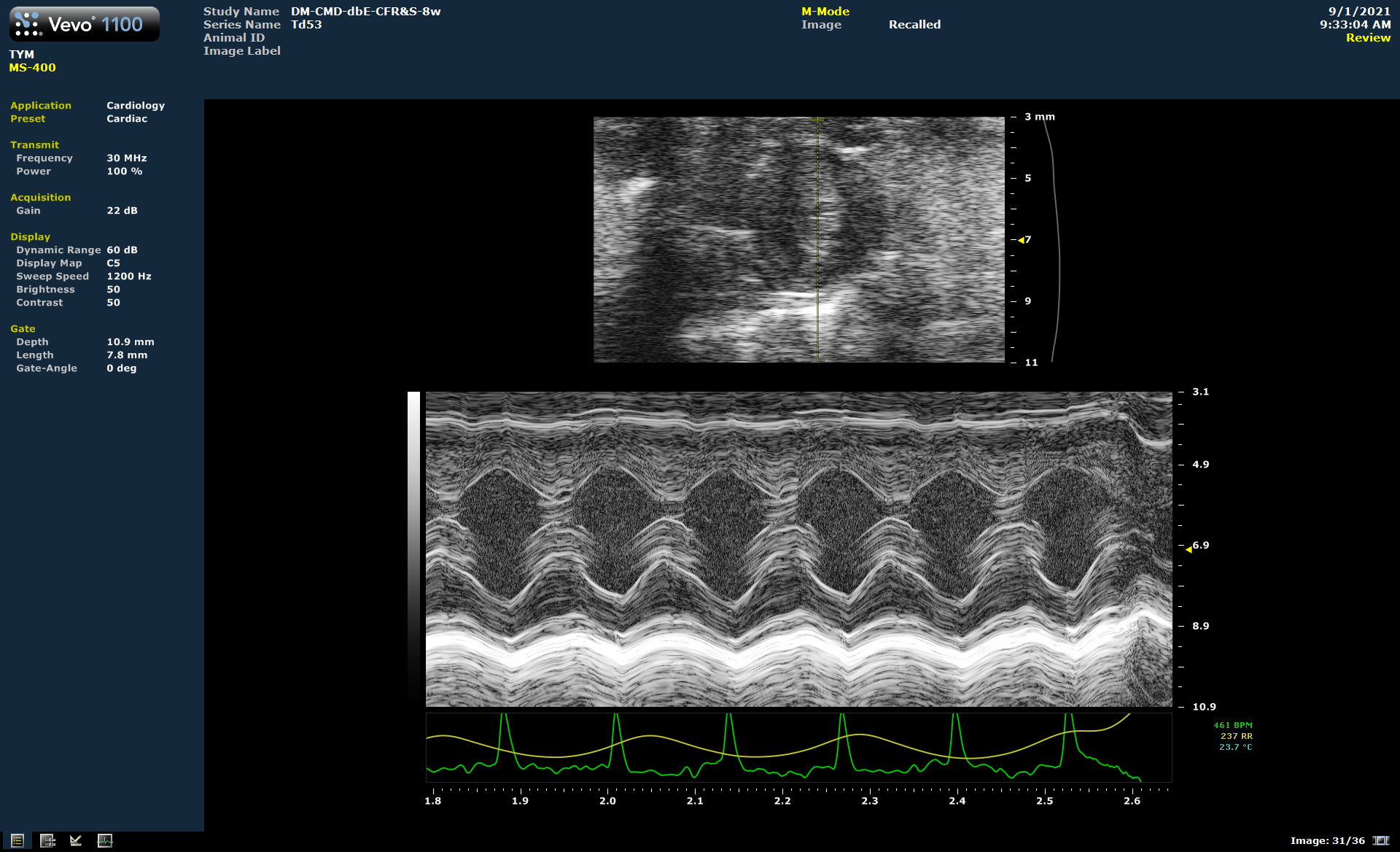

Supplement: Supplementary file 3 [file Data_Sheet_1.ZIP › 0801σÄƒσoïμò░μì«20221124Σ┐«μö╣/Figure2/Cardiac systolic function/db:db+EMPA-5-systolic.tif]

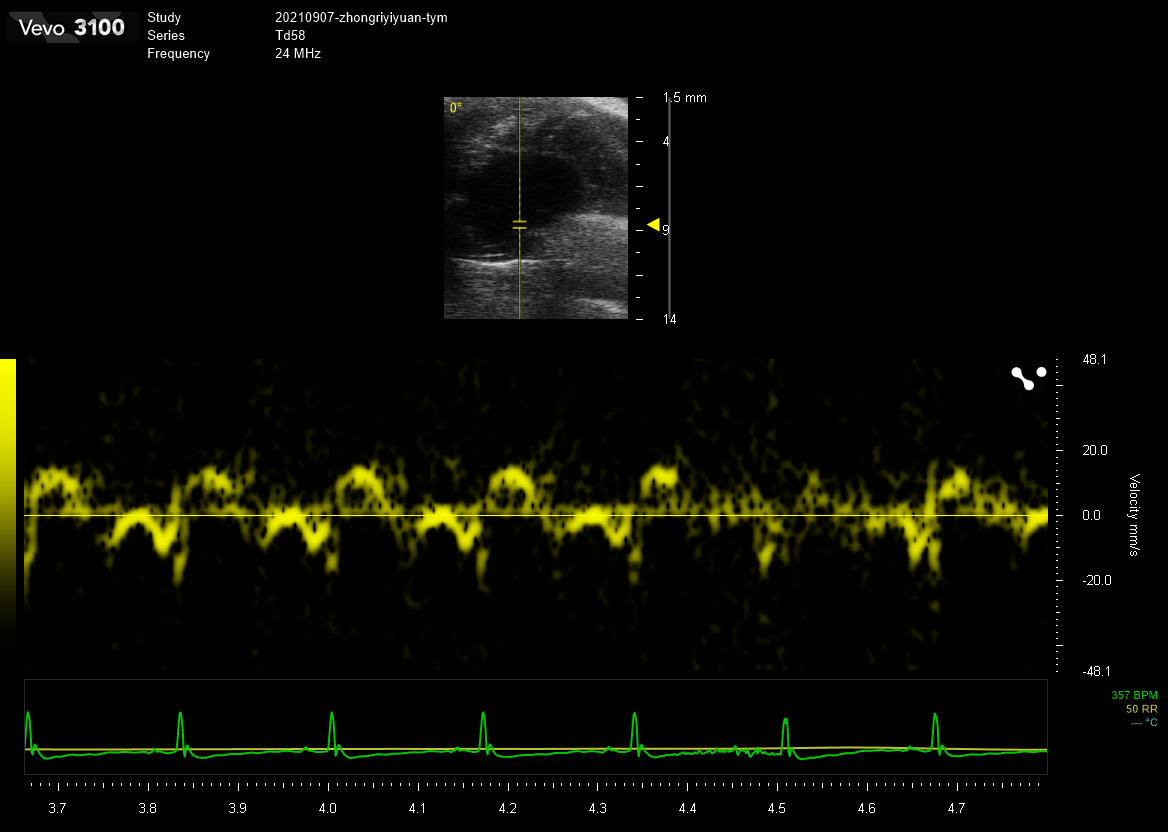

Supplement: Supplementary file 3 [file Data_Sheet_1.ZIP › 0801σÄƒσoïμò░μì«20221124Σ┐«μö╣/Figure2/E:e'/db:db_2021-09-07-11-16-33.tif]

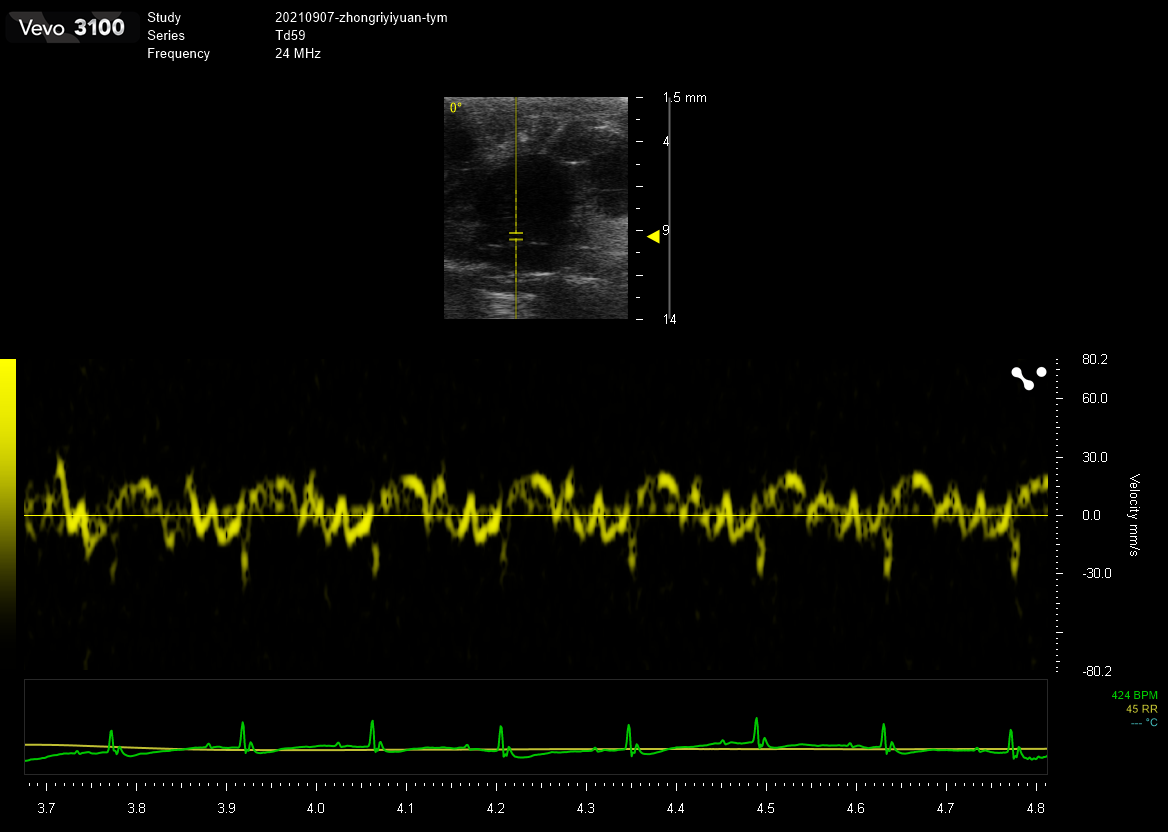

Supplement: Supplementary file 3 [file Data_Sheet_1.ZIP › 0801σÄƒσoïμò░μì«20221124Σ┐«μö╣/Figure2/E:e'/db:db+EMPA_2021-09-07-11-02-05.tif]

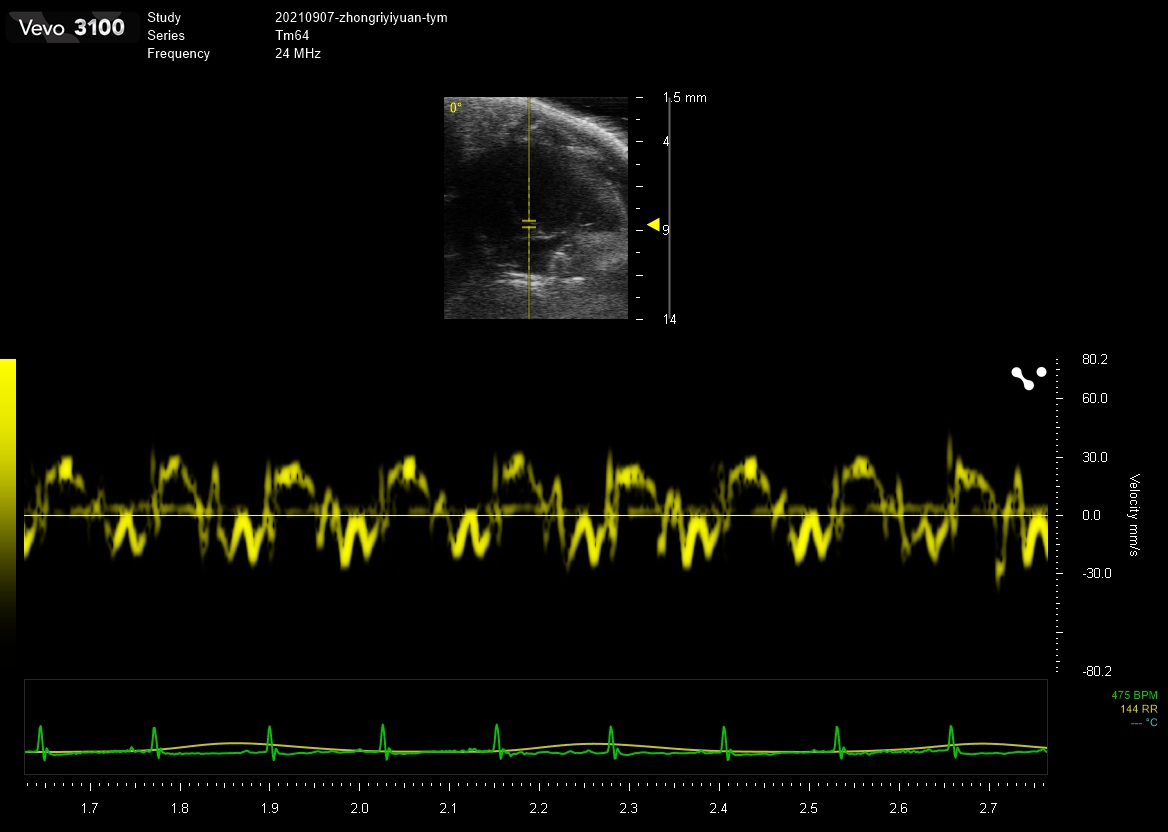

Supplement: Supplementary file 3 [file Data_Sheet_1.ZIP › 0801σÄƒσoïμò░μì«20221124Σ┐«μö╣/Figure2/E:e'/db:m_2021-09-07-11-24-45.tif]

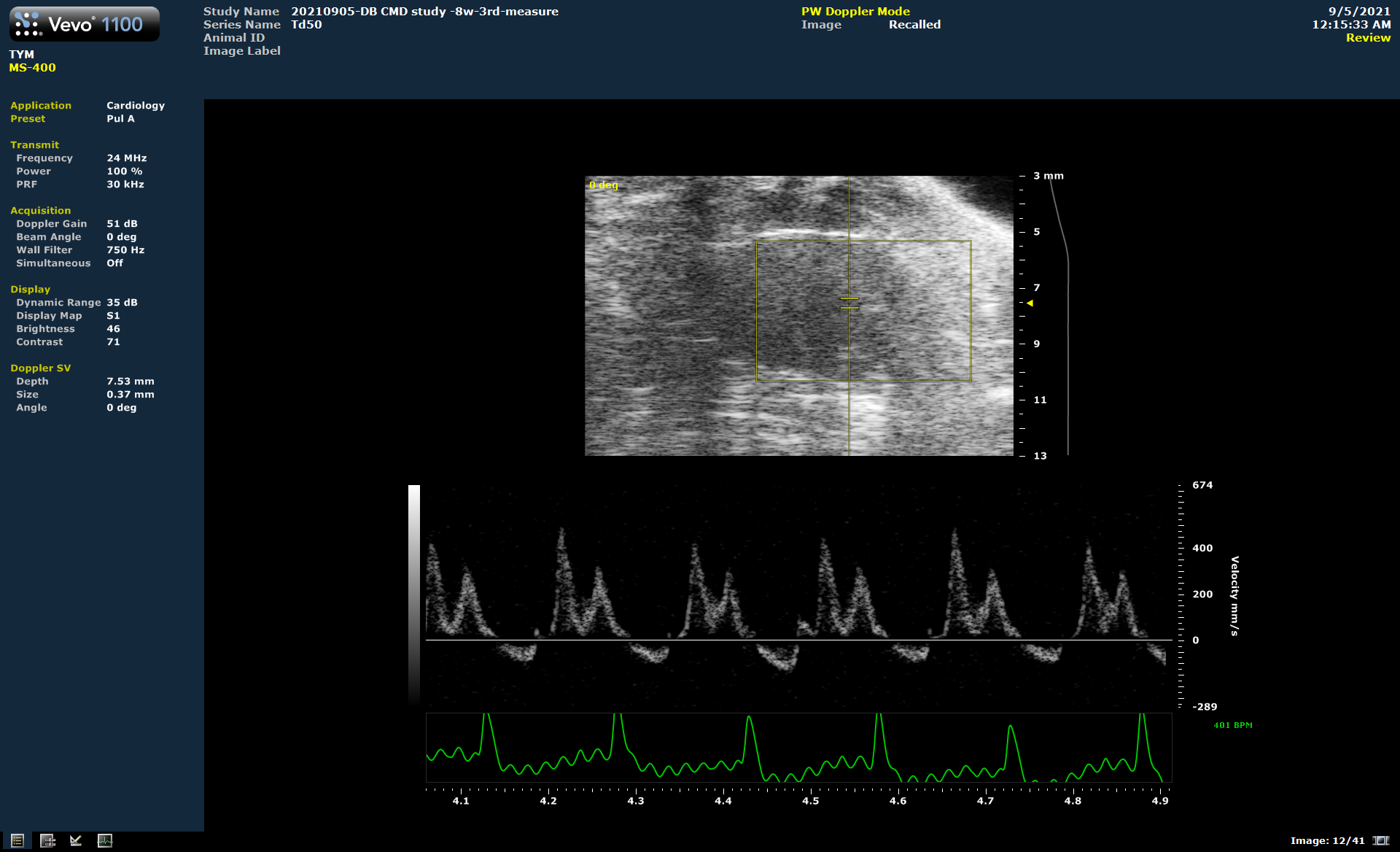

Supplement: Supplementary file 3 [file Data_Sheet_1.ZIP › 0801σÄƒσoïμò░μì«20221124Σ┐«μö╣/Figure2/Cardiac diastolic function/db:db+EMPA-4-diastolic.tif]

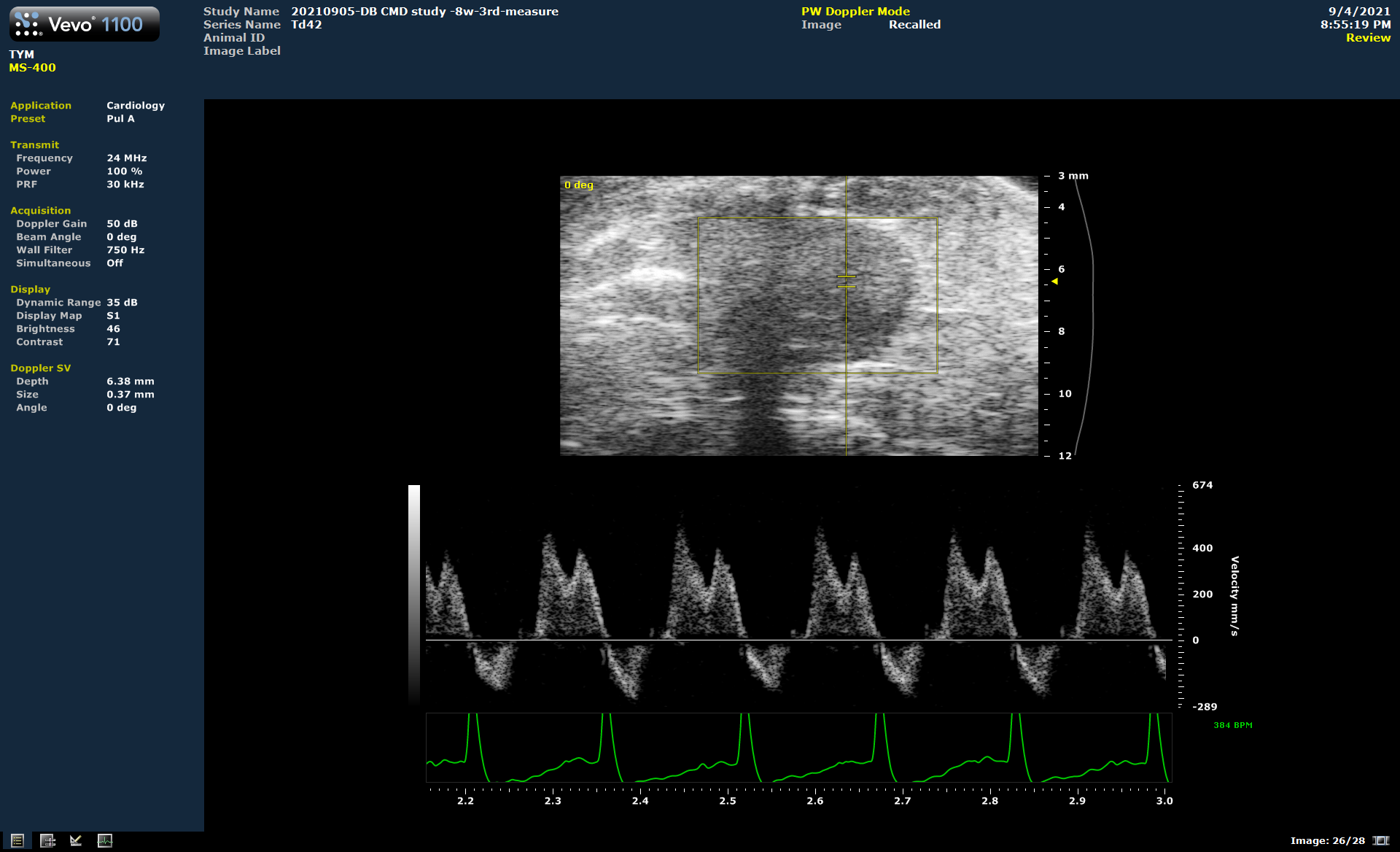

Supplement: Supplementary file 3 [file Data_Sheet_1.ZIP › 0801σÄƒσoïμò░μì«20221124Σ┐«μö╣/Figure2/Cardiac diastolic function/db:db-2-diastolic.tif]

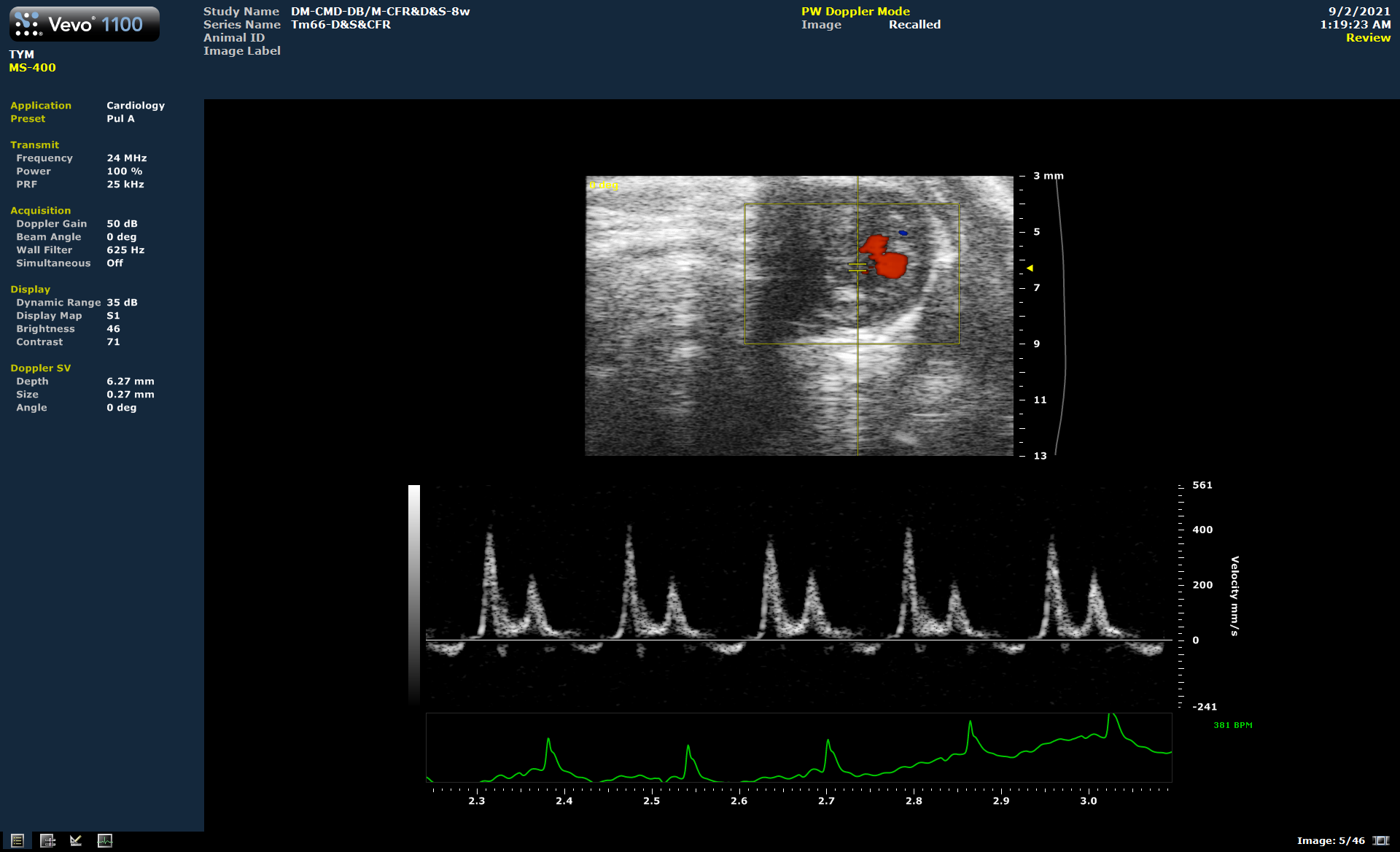

Supplement: Supplementary file 3 [file Data_Sheet_1.ZIP › 0801σÄƒσoïμò░μì«20221124Σ┐«μö╣/Figure2/Cardiac diastolic function/db:m-5-diastolic.tif]

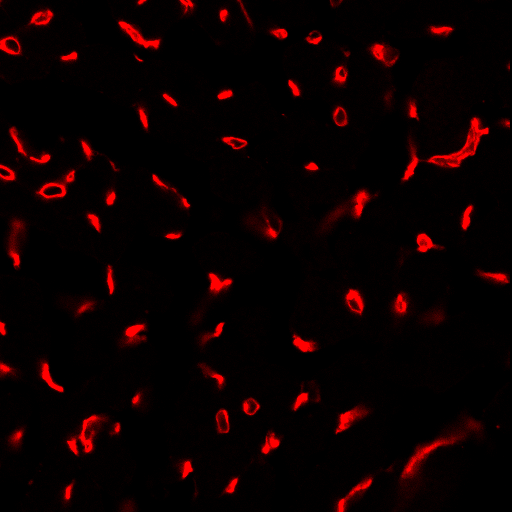

Supplement: Supplementary file 3 [file Data_Sheet_1.ZIP › 0801σÄƒσoïμò░μì«20221124Σ┐«μö╣/Figure4/Capillary number/db:db-8-LV-IB4-x40.tif]

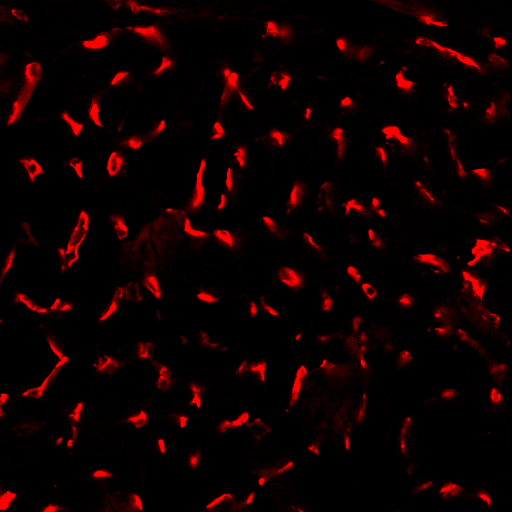

Supplement: Supplementary file 3 [file Data_Sheet_1.ZIP › 0801σÄƒσoïμò░μì«20221124Σ┐«μö╣/Figure4/Capillary number/db:db+EMPA-3-LV-IB4-x40.tif]

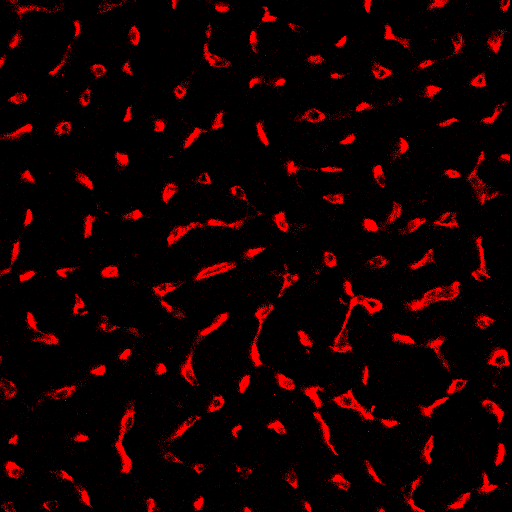

Supplement: Supplementary file 3 [file Data_Sheet_1.ZIP › 0801σÄƒσoïμò░μì«20221124Σ┐«μö╣/Figure4/Capillary number/db:m-4-LV-IB4-x40.tif]
